# Understanding of Various Type of Unambiguous Discrimination in View of Coherence Distribution

**DOI:** 10.3390/e22121422

**Published:** 2020-12-16

**Authors:** Min Namkung, Younghun Kwon

**Affiliations:** 1 School of Applied Mathematics, National Research University Higher School of Economics, 101000 Moscow, Russia; mslab.nk@gmail.com; 2 Department of Applied Physics, Hanyang University, Ansan, Kyunggi-Do 425-791, Korea

**Keywords:** coherence distribution, generalized measurement, unambiguous discrimination, sequential state discrimination, assisted optimal state discrimination

## Abstract

Unambiguous quantum state discrimination is a strategy where the conclusive result can always be trusted. This strategy is very important, since it can be used for various quantum information protocols, including quantum key distribution. However, in the view of quantumness, it is not clear what is going on in performing unambiguous quantum state discrimination. To answer the question, we investigate coherence distribution when unambiguous discrimination is performed by generalized measurement. Specially, we study coherence distribution in three cases, which consist of unambiguous quantum state discrimination, sequential quantum state discrimination, and assisted optimal discrimination, which are considered to be a family of unambiguous quantum state discrimination. In this investigation, we show that the structure of generalized measurements performing various types of unambiguous quantum state discrimination can be understood in terms of coherence distribution. Our result is not limited to the discrimination of two pure quantum states, but it is extended to the discrimination of two mixed states.

## 1. Introduction

Unambiguous quantum state discrimination (**UD**) is a strategy for discriminating quantum states without an error. The errorless results in **UD** require of containing the inconclusive result. Even though many successful applications of **UD** have been known, it is not clear what can make **UD** successful. It is known that **UD** can be constructed in terms of generalized measurement [1,2,3,4,5,6,7]. Because generalized measurement performing **UD** should consist of preparing an auxiliary(or ancilla) system and interacting with a given quantum system [8,9], understanding what is going on in the interaction of **UD** is very important.

One can regard **UD** as a game between a sender called Alice and a receiver called Bob. When the interaction is terminated, there can be many choices for Bob in generalized measurement, according to whether Bob’s measurement is optimal or not and which systems Bob measures on. The first choice is that Bob only performs a local projective measurement on his auxiliary system. If Bob performs non-optimal unambiguous discrimination, which means that Bob’s measurement does not optimize average success probability, then one can obtain partial information from Bob’s post-measurement state [10]. It leads to sequential state discrimination (**SSD**) [10,11,12,13,14,15,16,17,18]. The second choice is that Bob performs local projective measurement on both Alice and his system. This choice leads to assisted optimal state discrimination (**AOSD**) [19,20,21,22]. In fact, **AOSD** is classified into two cases, such as **AOSD1** and **AOSD2**. In **AOSD1**, Bob discriminates one out of Alice’s two quantum states. Meanwhile, in **AOSD2**, Bob discriminates every Alice’s quantum state.

Quantum coherence [23] is an important feature in revealing the quantum nature of a system. It is known to help us to understand the concept of wave-particle duality [24,25,26]. Not only that, it is suggested that quantum coherence can be related to a various quantum information processings [27,28,29,30,31]. Additionally, quantum coherence has an advantage in that it can be studied in a single system as well as in a multipartite system. Further, it can be used to unify various quantum characteristics. Additionally, it can help to understand the behavior of quantum operation [32].

Therefore, in this work, using coherence, we study what is going on when the generalized measurement is performed for various types of **UD**. To do it, we investigate how coherence is distributed in performing generalized measurements for **UD**, **SSD**, and **AOSD**. For the purpose, we consider relative quantum coherence (RQC), as a coherence measure [33]. Further, in order to understand the behavior of coherence distribution, we need to study the localization of relative quantum coherence (RQC). For the measure of localization of relative quantum coherence (RQC), we consider entropic quantum discord [34,35,36,37,38,39,40] and symmetrized discord [11,15].

Our investigation tells that the unitary operator in generalized measurement for **UD** moves the localization of RQC in Alice’s initial ensemble(or average state) into Bob’s auxiliary system. Meanwhile, the unitary operator in generalized measurement for **SSD** distributes RQC in Alice’s initial ensemble to both Alice’s and Bob’s systems. Further, the unitary operator in generalized measurement for **AOSD1** moves the localization of RQC in Alice’s initial ensemble into Bob’s auxiliary system. Meanwhile, the unitary operator in generalized measurement for **AOSD2** distributes RQC in Alice’s initial ensemble to both Alice’s and Bob’s systems.

In an operational view, our result implies that coherence distribution depends on the way where Bob constructs his generalized measurement. As known, **UD**, **SSD**, **AOSD1**, and **AOSD2** performs unambiguous discrimination. Because coherence is regarded as a resource for quantum operation, understanding how coherence distribution occurs in these scenarios is an important subject of investigation. We show that the feature of coherence distribution in these scenarios depends on the characteristic of each scenario’s measurement. Further, we show that our argument could be extended to cases, including two mixed states. In other words, our arguments listed above is not limited to discrimination of two pure states. In terms of entropic quantum discord and symmetrized discord as witnesses of RQC localization, our result can help to understand the quantum correlations.

Furthermore, our result consistently reveals a relation between quantumness and unambiguous quantum state discrimination. It is well known that **AOSD** requires partially quantum dissonance, which is defined as quantum discord in separable state [41]. Meanwhile, our result explains the relationship between coherence distribution and **AOSD**. Additionally, it should be noted that we could extend our argument successfully to two mixed states case.

This paper is organized. as follows. In Section 2, we briefly review RQC and RQC localization. Additionally, we show that entropic quantum discord and symmetrized discord can be applied for witnesses of RQC localization. In Section 3, we explain four strategies (**UD**, **SSD**, **AOSD1**, and **AOSD2**), which can be understood as a deformed structure of **UD**. (Actually, **AOSD1** and **AOSD2**) have an equivalent structure as **AOSD**. In Section 4, we analyze coherence distribution in generalized measurements for four cases when every two quantum state of Alice is pure. In Section 5, we extend our argument in Section 4 to mixed states case. Finally, we conclude and discuss our result in Section 6. Because **UD** based algorithms can be applied to quantum random number generation [42], quantum key distribution [43,44], and quantum state tomography [45], our result can contribute to understanding how coherence distribution is essential in quantum information protocols.

## 2. Preliminaries

### 2.1. Definition of RQC

Although superposition is one of the fundamental concepts in quantum physics, it is difficult to rigorously define the notion of superposition. Despite this, the state of a system without superposition is expressed as a classical probability distribution, which is equivalent to the diagonal density operator. This state is known as the incoherent state. Additionally, the incoherent operation is defined as a completely positive map (CP map), which maps the set of incoherent state onto itself. In this way, coherence can be defined as a quantity in which incoherent operation does not increase.

From this, the mathematical conditions of coherence measure C(·) can be provided, as follows [23]:

(C1) Assume that I is the set of incoherent states. Then, C(δ)=0 if and only if δ∈I.
(C2) Assume that incoherent operation is expressed as a set of Kraus operators $\left\{K_{1},\cdots ,K_{n}|K_{i}IK_{i}^{†}\subset I\;\;\forall i\right\}$.
  −Given the incoherent CPTP map, *C* satisfies C(ρ)≥C(Φ(ρ)), where $\Phi \left(\rho \right)=\sum_{i=1}^{n}K_{i}\rho K_{i}^{†}$.
  −For the post-measurement state $\rho_{i}=K_{i}\rho K_{i}^{†}/p_{i}$ and the corresponding probability $p_{i}=\mathrm{Tr}\left[K_{i}\rho K_{i}^{†}\right]$, *C* satisfies $C\left(\rho \right)\geq \sum_{i=1}^{n}p_{i}C\left(\rho_{i}\right)$.
(C3) *C* is convex, meaning that *C* satisfies $\sum_{i=1}^{n}p_{i}C\left(\rho_{i}\right)\geq C\left(\sum_{i=1}^{n}p_{i}\rho_{i}\right)$.

According to Baumgratz, Cramer, and Plenio [23], the relative entropy of coherence satisfies the above three conditions and it is defined as

$$\begin{array}{c} C_{\mathrm{rel}.\mathrm{ent}}\left(\rho \right)=S\left(\rho_{\mathrm{diag}}\right)-S\left(\rho \right). \end{array}$$

**Figure 1:**
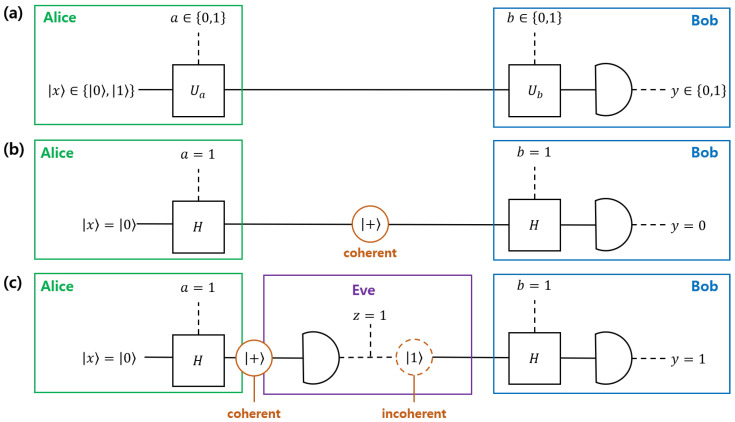
Description of BB84 protocol and coherence therein. In (**a**), Alice prepares a qubit |x〉∈{0,1}, and Bob performs a projective measurement {|0〉〈0|,|1〉〈1|}. Here, Alice (Bob) performs a unitary operator $U_{a}$ ($U_{b}$), depending on a random bit a∈{0,1} (b={0,1}). Here, $U_{x}$ is an identity if x=0, and $U_{x}$ is a Hadamard if x=1. In (**b**), we assume that Alice prepares |x〉=|0〉, and *a* is given as a=1. Subsequently, $U_{a}=H$ transforms |0〉 into |+〉, which has nonzero coherence. If b=1, Bob obtains a measurement outcome y=0 which is same as x=0. In (**c**), Eve appears between Alice and Bob. Eve uses a strategy to measure Alice’s qubit and sends |z〉, which corresponds to her outcome *z*, to Bob. Suppose that x=0 and a=1. Subsequently, Eve’s operation can be incoherent, since it transforms the coherent state |+〉 into the noncoherent state, on a fixed basis. Because of this, there is a possibility that Bob obtains y=1, even if x=0.

**Example** **1.** *(Role of coherence in BB84 protocol) Most of quantum key distribution protocol uses a superposed state as an information carrier, which has nonzero coherence, in order to provide security between a sender and a receiver. Here, we introduce a BB84 protocol [43], which is described in Figure 1. In Figure 1a, Alice produces a qubit |x〉∈{|0〉,|1〉}, and performs a unitary operator $U_{a}$, depending on her random bit a∈{0,1}. Here, $U_{a}$ is an identity if a=0 and a Hadamard gate if a=1. Subsequently, Alice sends one out of four qubits {|0〉,|1〉,|+〉,|−〉}. After Alice sends a qubit to Bob, Bob performs a unitary operator $U_{b}$, depending on his random bit b∈{0,1}. Here, $U_{b}$ is an identity if b=0 and a Hadamard gate if b=1. Subsequently, Bob performs a projective measurement {|0〉〈0|,|1〉〈1|} and obtains a measurement outcome of y∈{0,1}.*

We assume that *a* and *b* are randomly choosen as a=1 and b=1, as in Figure 1b. Additionally, we assume that Alice prepares a qubit |0〉. Subsequently, Alice’s unitary operator $U_{a=1}=H$ transforms |0〉 into |+〉, which has nonzero coherence in the fixed basis {|0〉,|1〉}. Because Bob’s unitary operator $U_{b=1}=H$ transforms |+〉 into |0〉, Bob always obtains the measurement outcome of y=0, when Alice prepares |0〉.

In Figure 1c, Eve performs eavesdropping between Alice and Bob. Here, we assume that Eve performs a strategy that, after performing her measurement, she sends the qubit |z〉 corresponding to the measurement outcome of z∈{0,1} to Bob. Note that Eve’s post-measurement state can be incoherent. Subsequently, there is a possibility that Bob obtains a measurement outcome of y=1, which is different from x=0. Thus, Alice and Bob can notice Eve’s presence by comparing their list of *a* and *b*.

Consequently, it implies that coherence can be a resource for security of BB84.

Additionally, M.-L. Hu and H. Fan [33] defined the basis-dependent measure of coherence as

$$\begin{array}{ccc} C_{\mathrm{rel}.\mathrm{ent}}\left(\rho ,\sigma \right) & = & -\sum_{i}\langle \psi_{i}\left|\rho \right|\psi_{i}\rangle \log_{2}\langle \psi_{i}\left|\rho \right|\psi_{i}\rangle -S\left(\rho \right). \end{array}$$

where ${\left\{|\psi_{i}\rangle \right\}}_{i}$ is a fixed basis, consisting of σ. This coherence measure is also known as the *relative quantum coherence(RQC)*. If σ is degenerate, then the basis that consists of σ is not unique [33]. Therefore, the suprenum of the RQC under every basis of σ is considered to be

$$\begin{array}{c} C_{\mathrm{rel}.\mathrm{ent}}^{\left(⋆\right)}\left(\rho ,\sigma \right)=\underset{{\left\{|\psi_{i}\rangle \right\}}_{i}}{\sup}C_{\mathrm{rel}.\mathrm{ent}}\left(\rho ,\sigma \right). \end{array}$$

This measure of coherence is also called the *maximum RQC* [33]. It is well known that RQC is applicable for bipartite system, where it can be related to various quantum correlations, including quantum discord, measurement-induced disturbance, and nonlocality. Particularly, entropic quantum discord has a close relationship with RQC localization.

### 2.2. Definition of RQC Discrepancy and Localization

In order to define the RQC of a bipartite state $\rho_{AB}$, the construction of a fixed basis for systems *A* and *B* is required. Assume that both the fixed orthonormal basis ${\left\{\Pi_{i}^{\left(P\right)}\right\}}_{i}$ and ${\left\{\Pi_{i}^{\left(Q\right)}\right\}}_{i}$ consist of each system *A* and *B*. The incoherent state $\rho_{PQ}$, which corresponds to $\rho_{AB}$, is then given as $\rho_{PQ}=\sum_{i,j}\Pi_{i}^{\left(P\right)}⊗\Pi_{j}^{\left(Q\right)}\rho_{AB}\Pi_{i}^{\left(P\right)}⊗\Pi_{j}^{\left(Q\right)}$. Likewise, the incoherent state $\rho_{P}$($\rho_{Q}$), which corresponds to the partial state $\rho_{A}$($\rho_{B}$), is given as $\rho_{P}={\mathrm{Tr}}_{Q}\rho_{PQ}$($\rho_{Q}={\mathrm{Tr}}_{P}\rho_{PQ}$). Therefore, the RQCs of the states $\rho_{AB}$, $\rho_{A}$, and $\rho_{B}$ are defined as [33]

$$\begin{array}{ccc} & & C_{\mathrm{rel}.\mathrm{ent}}\left(\rho_{AB},\rho_{PQ}\right)=S\left(\rho_{PQ}\right)-S\left(\rho_{AB}\right),\\ & & C_{\mathrm{rel}.\mathrm{ent}}\left(\rho_{A},\rho_{P}\right)=S\left(\rho_{P}\right)-S\left(\rho_{A}\right),\\ & & C_{\mathrm{rel}.\mathrm{ent}}\left(\rho_{B},\rho_{Q}\right)=S\left(\rho_{Q}\right)-S\left(\rho_{B}\right). \end{array}$$

Because RQC is not increased under the trace out, $C_{\mathrm{rel}.\mathrm{ent}}\left(\rho_{AB},\rho_{PQ}\right)$ must be greater than or equal to both $C_{\mathrm{rel}.\mathrm{ent}}\left(\rho_{A},\rho_{P}\right)$ and $C_{\mathrm{rel}.\mathrm{ent}}\left(\rho_{B},\rho_{Q}\right)$. From an operational viewpoint, not every bipartite system $\rho_{AB}$ is guaranteed to be localized into a certain subsystem localized. In order to deal with this argument quantitively, the nonlocalized quantities of RQC are defined as

$$\begin{array}{ccc} \delta_{A}\left(\rho_{AB}\right) & \equiv & C_{\mathrm{rel}.\mathrm{ent}}\left(\rho_{AB},\rho_{PQ}\right)-C_{\mathrm{rel}.\mathrm{ent}}\left(\rho_{A},\rho_{P}\right),\\ \delta_{B}\left(\rho_{AB}\right) & \equiv & C_{\mathrm{rel}.\mathrm{ent}}\left(\rho_{AB},\rho_{PQ}\right)-C_{\mathrm{rel}.\mathrm{ent}}\left(\rho_{B},\rho_{Q}\right). \end{array}$$

$\delta_{A}$($\delta_{B}$) is also known as the RQC discrepancy. If $\delta_{A}$($\delta_{B}$) is equal to zero, $C_{\mathrm{rel}.\mathrm{ent}}\left(\rho_{AB},\rho_{PQ}\right)$ is equal to $C_{\mathrm{rel}.\mathrm{ent}}\left(\rho_{A},\rho_{P}\right)$($C_{\mathrm{rel}.\mathrm{ent}}\left(\rho_{B},\rho_{Q}\right)$). This means that the RQC of $\rho_{AB}$ is localized into system *A*(*B*). However, if $\delta_{A}$($\delta_{B}$) is nonzero, then the RQC of $\rho_{AB}$ is not localized into system *A*(*B*).

### 2.3. Entropic Quantum Discord as a Witness of RQC Localization

According to a study that was scarried out by M.-L. Hu and H. Fan [33], the discrepancy in the RQC is lower bounded by entropic quantum discord [34]

(1) $$\begin{array}{c} D_{A}\left(\rho_{AB}\right)\leq \delta_{A}\left(\rho_{AB}\right),\;\;D_{B}\left(\rho_{AB}\right)\leq \delta_{B}\left(\rho_{AB}\right). \end{array}$$

where $D_{A}\left(\rho_{AB}\right)$ and $D_{B}\left(\rho_{AB}\right)$ describe the entropic quantum discord, which is defined as

(2) $$\begin{array}{ccc} D_{A}\left(\rho_{AB}\right) & = & I\left(\rho_{AB}\right)-\max_{{\left\{M_{k}^{\left(A\right)}\right\}}_{k=1}^{N_{A}}}J\left({\left\{M_{k}^{\left(A\right)}\right\}}_{k=1}^{N_{A}}|B\right),\\ D_{B}\left(\rho_{AB}\right) & = & I\left(\rho_{AB}\right)-\max_{{\left\{M_{k}^{\left(B\right)}\right\}}_{k=1}^{N_{B}}}J\left({\left\{M_{k}^{\left(B\right)}\right\}}_{k=1}^{N_{B}}|A\right). \end{array}$$

Here, $I(\rho_{AB})$ is the von Neumann mutual information between systems *A* and *B*. Additionally, $J({\left\{M_{k}^{\left(A\right)}\right\}}_{k=1}^{N_{A}}|B)$($J({\left\{M_{k}^{\left(B\right)}\right\}}_{k=1}^{N_{B}})|A$) is the classical part of the mutual information between system *A* and *B*, where the local measurement that is expressed as POVM ${\left\{M_{k}^{\left(A\right)}\right\}}_{k=1}^{N_{A}}$(${\left\{M_{k}^{\left(B\right)}\right\}}_{k=1}^{N_{B}}$) is performed on system *A*(*B*) [46]. Equation (1) implies that entropic quantum discord is applicable as a witness for RQC localization. If the entropic quantum discord $D_{A}\left(\rho_{AB}\right)$($D_{B}\left(\rho_{AB}\right)$) is nonzero, then the RQC of $\rho_{AB}$ is not localized into system *A*(system *B*).

The witness in Equation (1) is asymmetric under a change in the lower index. In order to investigate RQC localization, the witness for system *A* and *B* should be simultaneously tested. Hence, if a witness is symmetrical under a change in the lower index, we can simultaneously investigate the RQC localization by testing one witness. Fortunately, symmetrized discord can be exploited [11]:

$$\begin{array}{c} D_{AB}\left(\rho_{AB}\right)=\sqrt{D_{A}\left(\rho_{AB}\right)D_{B}\left(\rho_{AB}\right)}, \end{array}$$

Substituting Equation (1) into Equation (2), an inequality is obtained as

$$\begin{array}{c} D_{AB}\left(\rho_{AB}\right)\leq \sqrt{\delta_{A}\left(\rho_{AB}\right)\delta_{B}\left(\rho_{AB}\right)}. \end{array}$$

If $D_{AB}\left(\rho_{AB}\right)$ is nonzero, the RQC of $\rho_{AB}$ is not localized into *A* or *B*. Moreover, if the RQC of $\rho_{AB}$ is localized into either system *A* or *B*, the symmetrized discord is equal to zero. Hence, the symmetrized discord can be applied as a witness in a similar that is manner to symmetric discord [33].

**Example** **2.** *(RQC localization during CNOT operation) By using the formula that is proposed in Ref. [36], the entropic quantum discord of the maximally entangled state is evaluated as $D_{A}\left(|\psi_{-}\rangle \langle \psi_{-}|\right)=D_{B}\left(|\psi_{-}\rangle \langle \psi_{-}|\right)=1$. Therefore, the symmetrized discord is nonzero. This implies that RQC of the maximally entangled state is not localized in either the system A or the system B. Additionally, the RQC of the maximally entangled state is $C_{rel.ent}\left(|\psi_{-}\rangle \langle \psi_{-}|,\rho_{PQ}\right)=1$, where the fixed basis is {|0〉,|1〉} and $\rho_{PQ}$ is 0.5|01〉〈01|+0.5|10〉〈10|.*

**Figure 2:**
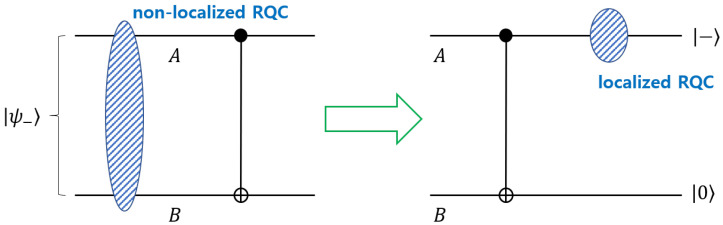
Relative quantum coherence (RQC) localization in CNOT operation. When the input is a maximally entangled state $|\psi_{-}\rangle =\left(|01\rangle -|10\rangle \right)\sqrt{2}$, RQC of it is not localized in either a system *A* or a system *B*. However, after the CNOT operation, the RQC is localized in a system *A*.

After CNOT operation, the composite state is transformed into |−0〉. Because this state is a product state, the entropic quantum discord is zero. This implies that the RQC is localized in either the system *A* or the system *B*. Because the RQC of the system *A* is evaluated as $C_{rel.ent}\left(|-\rangle \langle -|,\rho_{P}\right)=1$, where $\rho_{P}$ is a maximally mixed state, we can conclude that the RQC of the maximally entangled state is localized in the system *A* by the CNOT gate. It implies that RQC localization may be understood as a resource for implementing CNOT operation (Figure 2).

## 3. Structure of Various Protocols Based on Unambiguous Discrimination

Suppose that unambiguous discrimination is performed by Alice and Bob. Subsequently, one can assume that Alice prepares a pure state $|\psi_{i}\rangle \in \{|\psi_{1}\rangle ,|\psi_{2}\rangle \}$, with a prior probability $q_{i}$. Here, $|\psi_{1}\rangle$ is not orthogonal to $|\psi_{2}\rangle$. Alice sends a pure state $|\psi_{i}\rangle$ to Bob through a quantum channel. Subsequently, Bob can discriminate Alice’s pure state without any error. Mathematically, Bob’s measurement is expressed as three elements POVM $\left\{M_{0},M_{1},M_{2}\right\}$. Here, $M_{i}$ is an element that corresponds to a measurement outcome i∈{0,1,2}. If i≠0, then Bob can conclude that Alice’s pure state is $|\psi_{i}\rangle$ without any error. In this case, the outcome of measurement *i* is considered as a conclusive outcome. Meanwhile, if i=0, Bob cannot conclude which of Alice’s pure states is prepared. In this case, the measurement outcome i=0 is considered as the inconclusive outcome.

If the possibility that Bob obtains an inconclusive outcome is excluded, then Bob cannot carry out the measurement which discriminates Alice’s pure states without an error. This is because nonorthogonal pure states cannot be perfectly discriminated, according to quantum theory.

When the generalized measurement is considered, Bob needs to extend the Hilbert space, where Alice’s pure state resides. If the Hilbert space of Alice (Bob) is denoted as $H_{A}$($H_{B}$), the task of generalized measurement should consider a unitary operator $U_{AB}:H_{A}⊗H_{B}\to H_{A}⊗H_{B}$, where Alice’s pure state resides in $H_{A}$ and the state of Bob’s auxiliary system is found in $H_{B}$.

After the unitary operator $U_{AB}$ is performed, Bob’s composite state is measured via a projective measurement. Bob then discriminates Alice’s pure states without any error, which is obtained by the projective measurement. The structure of $U_{AB}$ depends on how Bob constructs the projective measurement.

### 3.1. The Structure of Unambiguous Discrimination (**UD**) and Sequential State Discrimination (**SSD**)

First, if Bob only performs a local projective measurement on his ancilla system, the structure of $U_{AB}$ is:

(3) $$\begin{array}{c} U_{AB}{|\psi_{i}\rangle }_{A}⊗{|b\rangle }_{B}=\sqrt{\alpha_{i}}{|\phi_{i}\rangle }_{A}⊗{|i\rangle }_{B}+\sqrt{1-\alpha_{i}}{|\phi_{0}\rangle }_{A}⊗{|0\rangle }_{B}. \end{array}$$

**Figure 3:**
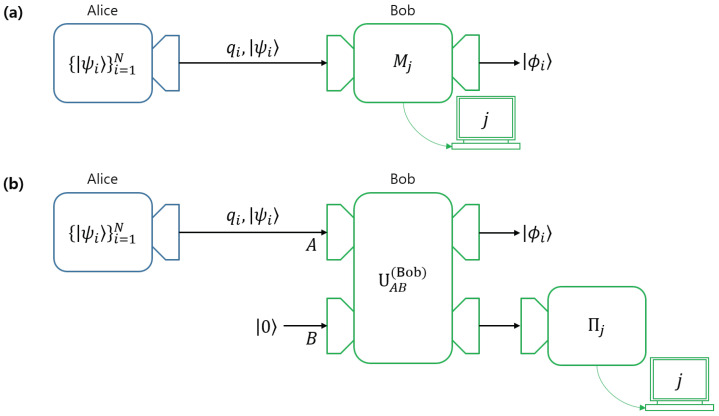
(**a**) Unambiguous discrimination between Alice and Bob, where Bob’s measurement is expressed as POVM $\left\{M_{0},M_{1},M_{2}\right\}$. (**b**) The structure of unambiguous quantum state discrimination (**UD**), where Bob performs a local projective measurement on his auxiliary system. In this structure, the unitary operator generates a post-measurement state that corresponds to a specific measurement outcome. If Bob carries out a non-optimal unambiguous discrimination, Charlie can discriminate Bob’s post-measurement states. In this case, (**b**) is obviously in the form of sequential state discrimination (**SSD**).

Bob’s local projective measurement is expressed as ${\left\{|i\rangle \langle i|\right\}}_{i=0}^{2}$. The non-negative real value $\alpha_{i}$ is the probability that Bob obtains the conclusive outcome *i*, and $1-\alpha_{i}$ is the probability that Bob obtains the inconclusive outcome (see Figure 3). A controlled-unitary operator $U_{c}$ performs $|\phi_{0}\rangle ⊗|0\rangle \to |\phi_{i}\rangle ⊗|0\rangle$ and $|\phi_{i}\rangle ⊗|i\rangle \to |\phi_{i}\rangle ⊗|i\rangle$. Therefore, we can consider $U_{AB}$ as $U_{c}U_{AB}\to U_{AB}$, without any loss of generality. Therefore, Equation (3) can also be expressed as

(4) $$\begin{array}{c} U_{AB}{|\psi_{i}\rangle }_{A}⊗{|b\rangle }_{B}={|\phi_{i}\rangle }_{A}⊗\left\{\sqrt{\alpha_{i}}{|i\rangle }_{B}+\sqrt{1-\alpha_{i}}{|0\rangle }_{B}\right\}. \end{array}$$

The necessary and sufficient condition for global unitary operator of Equations (3) and (4) can be obtained by the following Lemma [47].

**Lemma** **1.** *Consider two sets $\left\{|\psi_{1}\rangle ,\cdots ,|\psi_{N}\rangle \right\}$ and $\left\{|\phi_{1}\rangle ,\cdots ,|\phi_{N}\rangle \right\}$, which consist of finite vectors. For ∀i∈{1,⋯,N}, the necessary and sufficient condition for global unitary operator performing $U|\psi_{i}\rangle =|\phi_{i}\rangle$ is that for ∀i,j∈{1,⋯,N}, one has $\langle \psi_{i}|\psi_{j}\rangle =\langle \phi_{i}|\phi_{j}\rangle$.*

Applying this Lemma to Equations (3) and (4), the necessary and sufficient condition for $U_{AB}$ and $U_{AB}$ is given, as follows:

(5) $$\begin{array}{c} \langle \psi_{i}|\psi_{j}\rangle =\sqrt{\left(1-\alpha_{i}\right)\left(1-\alpha_{j}\right)}\langle \phi_{i}|\phi_{j}\rangle . \end{array}$$

In other words, the strategy of quantum state discrimination when considering Equations (3) and (4) has the identical constraint. This implies that, without losing consistency, one can choose the formalism of Equation (4). (If the post-measurement state of Bob is given by one of the set $\left\{|\phi_{1}\rangle ,|\phi_{2}\rangle \right\}$, then Charlie can discriminate the post-measurement state of Bob without any error. Therefore, the formalism of Equation (4) provides sequential state discrimination (**SSD**) of Bob and Charlie.)

Suppose that Alice prepares one of two pure states $|\psi_{1}\rangle ,|\psi_{2}\rangle$. Here, let us assume that $\langle \psi_{1}|\psi_{2}\rangle$ is a real number. Subsequently, the explicit form of $U_{AB}$ is expressed as [11]

$$\begin{array}{c} U_{AB}=\frac{1}{1-s^{2}}\left(|\phi_{1}\rangle {\langle {\tilde{\psi }}_{1}|}_{A}⊗|\eta_{1}\rangle {\langle b|}_{B}+|\phi_{2}\rangle {\langle {\tilde{\psi }}_{2}|}_{A}⊗|\eta_{2}\rangle {\langle b|}_{B}\right)+V. \end{array}$$

Here, $s=\langle \psi_{1}|\psi_{2}\rangle$, $|{\tilde{\psi }}_{i}\rangle =|\psi_{i}\rangle -|\psi_{j}\rangle \langle \psi_{j}|\psi_{i}\rangle$ and $|\eta_{i}\rangle =\sqrt{\alpha_{i}}|i\rangle +\sqrt{1-\alpha_{i}}|0\rangle$. V is an operator, which acts on the subspace of {|1〉,|2〉}. This explicitly means that $V{|\psi_{i}\rangle }_{A}⊗{|b\rangle }_{B}=0$ for all i∈{1,2}. When Equation (5) is satisfied, the structure of $U_{AB}$ provides various explicit forms of two input state $|\psi_{1}\rangle ,|\psi_{2}\rangle$ and post-measurement states $|\phi_{1}\rangle ,|\phi_{2}\rangle$. For instance, an explicit form of $|\psi_{1}\rangle ,|\psi_{2}\rangle$ can be given by

$$\begin{array}{c} |\psi_{j}\rangle =\sqrt{\frac{1+s}{2}}|0\rangle +{\left(-1\right)}^{j+1}\sqrt{\frac{1-s}{2}}|1\rangle . \end{array}$$

Afterwards, the explicit form of post-measurement states is provided by

(6) $$\begin{array}{c} |\phi_{j}\rangle =\sqrt{\frac{1+s^{\prime }}{2}}|0\rangle +{\left(-1\right)}^{j+1}\sqrt{\frac{1-s^{\prime }}{2}}|1\rangle ,s^{\prime }=\frac{s}{\sqrt{\left(1-\alpha_{1}\right)\left(1-\alpha_{j}\right)}}. \end{array}$$

When explicit forms of input states and post-measurement states are provided, $U_{AB}$ is determined. In **UD**, Bob discriminates pure states of Alice optimally. Therefore, the post-measurement states of Bob are completely overlapped ($s^{\prime }=1$). According to explicit form of Equation (6), the post-measurement states become $|\phi_{1}\rangle =|\phi_{2}\rangle =|0\rangle$, which are identical. (Suppose that $|\phi_{1}\rangle =|\phi_{2}\rangle =|+\rangle$. Because there exists an incoherent unitary operator performing |+〉→|0〉, the explicit form of post-measurement states cannot be fixed. In fact, the incoherent unitary operator acts on Alice’s system and it does not affect the optimal success probability and optimal conditions.) This implies that, in **UD**, the coherence of Alice’s ensemble is localized in Bob’s ancilla system.

In Equation (4), the unitary operator $U_{AB}$ generates a post-measurement state $|\phi_{i}\rangle$, which corresponds to the measurement outcome *i*. Therefore, there is a subsequent measurement, which can extract an information from Bob’s post-measurement state, if Bob performs a nonoptimal unambiguous discrimination. If Charlie constructs a subsequent measurement, Alice, Bob and Charlie can perform sequential state discrimination [10]. In this case, Charlie’s measurement includes the unitary operator $U_{AC}:H_{A}⊗H_{C}\to H_{A}⊗H_{C}$, which is expressed as

(7) $$\begin{array}{c} U_{AC}{|\phi_{i}\rangle }_{A}⊗{|c\rangle }_{C}={|\chi_{i}\rangle }_{A}⊗{\left\{\sqrt{\alpha_{i}^{\prime }}{|i\rangle }_{C}+\sqrt{1-\alpha_{i}^{\prime }}|0\rangle \right\}}_{C}. \end{array}$$

Charlie’s local projective measurement is expressed as ${\left\{|i\rangle \langle i|\right\}}_{i=0}^{2}$. The non-negative real value $\alpha_{i}^{\prime }$ is the probability that Charlie obtains a conclusive result *i*, and $1-\alpha_{i}^{\prime }$ is the probability that Charlie obtains a inconclusive result. The structure of sequential state discrimination (**SSD**) consists of $U_{AB}$ in Equation (4) and $U_{AC}$ in Equation (7). The structure of Charlie’s measurement depends on the setting of Bob’s measurement [10]. From Bob’s viewpoint, whether his measurement has structure of **UD** or **SSD** depends only on whether he performs an optimal discrimination or not. Because **UD** does not include Charlie, Bob has to optimally discriminate Alice’s states. However, in **SSD**, Bob should not perform optimal discrimination. If Bob performs the optimal unambiguous discrimination, then Charlie cannot perform unambiguous discrimination on Bob’s post-measurement states.

### 3.2. Structure of Assisted Optimal State Discrimination (**AOSD**)

Second, if Bob performs a projective measurement on both Alice’s system and his own, the unitary operator $V_{AB}$ is in the form [22]

$$\begin{array}{ccc} V_{AB}{|\psi_{1}\rangle }_{A}⊗{|0\rangle }_{B} & = & \sqrt{1-|\alpha_{1}{|}^{2}}{|0\rangle }_{A}⊗{|0\rangle }_{B}+\alpha_{1}{|\Phi \rangle }_{A}⊗{|1\rangle }_{B},\\ V_{AB}{|\psi_{2}\rangle }_{A}⊗{|0\rangle }_{B} & = & \sqrt{1-|\alpha_{2}{|}^{2}}{|1\rangle }_{A}⊗{|0\rangle }_{B}+\alpha_{2}{|\Phi \rangle }_{A}⊗{|1\rangle }_{B}, \end{array}$$

where {|0〉,|1〉} is the orthonormal basis which consists of $H_{A}$ and $H_{B}$. It is also apparent that $|\Phi \rangle =\cos\beta |0\rangle +\sin\beta e^{i\delta }|1\rangle$. Here, the inner product $\langle \psi_{1}|\psi_{2}\rangle$ is expressed as $\alpha_{1}^{*}\alpha_{2}=\left|\langle \psi_{1}|\psi_{2}\rangle \right|e^{i\delta }$.

**Figure 4:**
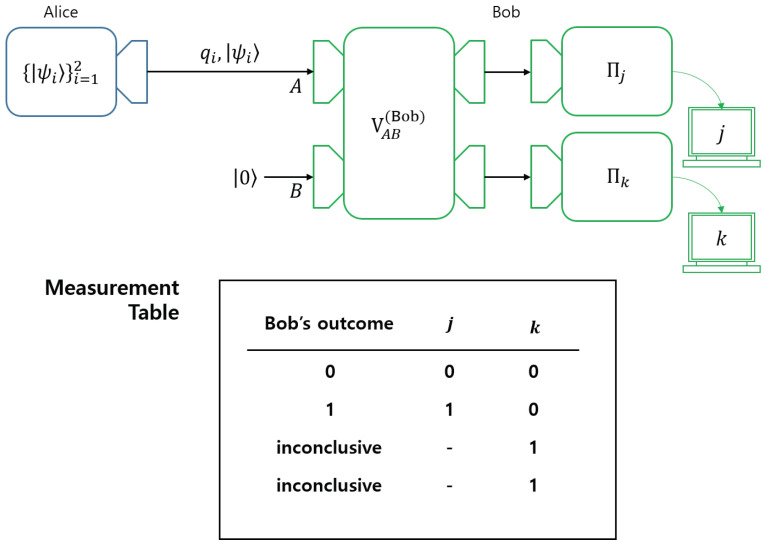
The structure of assisted optimal state discrimination (**AOSD**), where using a projective measurement, Bob measures both his system and Alice’s system. After the unitary operation is terminated, Bob performs local projective measurements on a system *A* and his auxiliary system *B*, respectively. Bob’s measurement outcome is expressed in the measurement table. Let each outcome of the projective measurements be denoted as *j* and *k*. If k=1, then Bob’s outcome is inconclusive, regardless of *j*. Meanwhile, if k=0, Bob can guess that Alice prepared $|\psi_{j}\rangle$ without an error.

Bob performs a local projective measurement ${\left\{|i\rangle \langle i|\right\}}_{i=0}^{1}$ on each system X∈{A,B} (See Figure 4). Here, $|\alpha_{i}|$ is the probability that Bob obtains the conclusive result *i*, and $\sqrt{1-|\alpha_{i}{|}^{2}}$ is the probability that Bob obtains the inconclusive result. If Bob obtains a conclusive result “0” by measuring system *B*, then he can discriminate Alice’s pure state, from the measurement outcome on system *A*. If the measurement outcome of system *A* is *i*, then, without any error, Bob can conclude that Alice’s pure state is $|\psi_{i}\rangle$. Meanwhile, if Bob obtains the conclusive result “1” by measuring system *B*, he cannot discriminate Alice’s pure states. Hence, the measurement outcome on system *B* informs Bob as to whether the measurement outcome is conclusive or not, and the result on system *A* tells Bob which pure state Alice has prepared.

This structure is known as assisted optimal state discrimination (**AOSD**). However, every case of **AOSD** uses both of the local projective measurements. Suppose that Bob discriminates one of Alice’s pure states(in this case, it is called **AOSD1**). Bob then only needs the measurement outcome on system *B*. Meanwhile, if Bob discriminates every pure state of Alice (in this case, it is called **AOSD2**), Bob should consider every measurement outcome.

## 4. Investigating Coherence Distribution in Various Protocols

### 4.1. Coherence Distribution in **UD** and **SSD**

Because the unitary operators of **UD** and **SSD** have the same structure, the coherence distribution of **UD** and **SSD** can be analyzed while using the same geometric structure. This geometric structure comes from the necessary and sufficient condition that the unitary operator $U_{AB}$ exists. Because $U_{AB}$ preserves the inner product between two pure states, the necessary and sufficient conditon that $U_{AB}$ exists is expressd as [14,16]

(8) $$\begin{array}{c} \left(1-\alpha_{1}\right)\left(1-\alpha_{2}\right)\geq s^{2}. \end{array}$$

**Figure 5:**
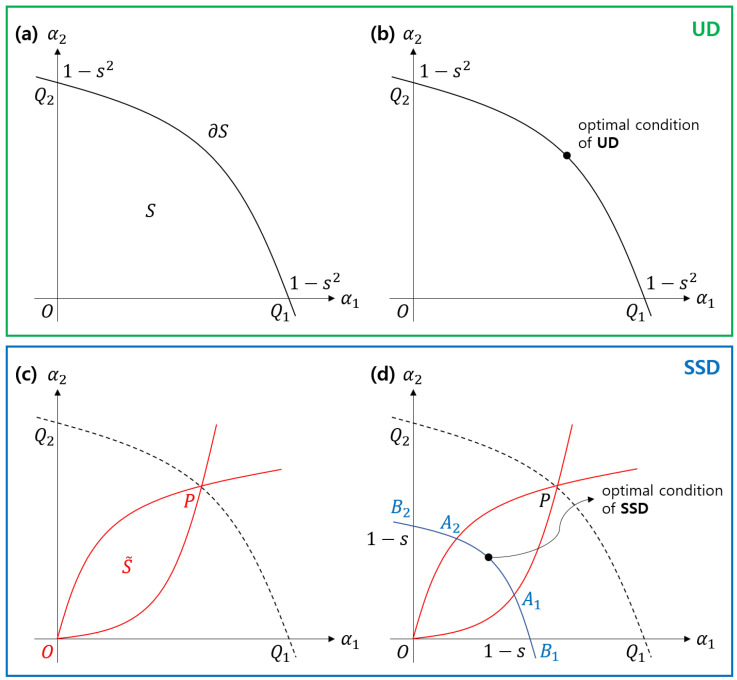
Geometric representation of the structures of **UD** and **SSD**. In (**a**), Bob’s POVM corresponds to a real vector $\left(\alpha_{1},\alpha_{2}\right)$ in a set S∪∂S, where *S* represents an interior of a circular sector $OQ_{1}Q_{2}$ and ∂S represents a curve $Q_{1}Q_{2}$ (solid black line). In (**b**), Bob’s POVM of optimal **UD** correponds to a real vector $\left(\alpha_{1},\alpha_{2}\right)$ on the ∂S. In (**c**), Bob’s POVM used for **SSD** corresponds to the interior or the surface of a closed convex set $\tilde{S}$ (solid red line). That is because, if $\left(\alpha_{1},\alpha_{2}\right)$ is included in $S-\tilde{S}$, one of Charlie’s POVM element is negative definite. In (**d**), Bob’s POVM of optimal **SSD** corresponds to a real vector $\left(\alpha_{1},\alpha_{2}\right)$ on the curve $B_{1}A_{1}A_{2}B_{2}$ (solid blue line).

Here, $s\equiv |\langle \psi_{1}|\psi_{2}\rangle |$. In Equation (8), it is assumed that, for post-measurement states $|\phi_{1}\rangle$ and $|\phi_{2}\rangle$, $|\langle \phi_{1}|\phi_{2}\rangle |\leq 1$. The set of two-dimensional real vectors $\left(\alpha_{1},\alpha_{2}\right)$, which satisfies Equation (8), can be geometrically expressed, as seen in Figure 5. Here, Bob’s measurement setting has one-to-one correspondence with a real vector $\left(\alpha_{1},\alpha_{2}\right)$. Additionally Bob’s optimal measurement corresponds to a tangential point between the curve $Q_{1}PQ_{2}$ and the line $P_{s}^{\left(B\right)}=q_{1}\alpha_{1}+q_{2}\alpha_{2}$ (here, $P_{s}^{\left(B\right)}$ is the average success probability of Bob).

In many discrimination strategies, Alice informs Bob of her prior probability distribution. This means that, from the viewpoint of Bob, Alice’s state is expressed as the initial ensemble (or average state) $q_{1}|\psi_{1}\rangle \langle \psi_{1}|+q_{2}|\psi_{2}\rangle \langle \psi_{2}|$ [11,22]. Therefore, the unitary operator $U_{AB}$ will produce an average state, expressed as

$$\begin{array}{ccc} \rho_{AB} & = & U_{AB}\left\{{\left(q_{1}|\psi_{1}\rangle \langle \psi_{1}|+q_{2}|\psi_{2}\rangle \langle \psi_{2}|\right)}_{A}⊗|b\rangle {\langle b|}_{B}\right\}U_{AB}^{†}\\ & = & \sum_{i\in \{1,2\}}q_{i}|\phi_{i}\rangle {\langle \phi_{i}|}_{A}⊗\left\{\sqrt{\alpha_{i}}|i\rangle +\sqrt{1-\alpha_{i}}|0\rangle \right\}{\left\{\sqrt{\alpha_{i}}\langle i|+\sqrt{1-\alpha_{i}}\langle 0|\right\}}_{B}. \end{array}$$

In **UD**, the post-measurement states become identical, which means that $|\phi_{1}\rangle =|\phi_{2}\rangle$. Therefore, $\rho_{A}\left(={\mathrm{Tr}}_{B}\rho_{AB}\right)$ is an incoherent state. Meanwhile, in **SSD**, $|\phi_{1}\rangle$ and $|\phi_{2}\rangle$ are neither orthogonal nor identical. Therefore, $\rho_{A}=q_{1}|\phi_{1}\rangle \langle \phi_{1}|+q_{1}|\phi_{2}\rangle \langle \phi_{2}|$ has nonzero coherence. Therefore, **UD** and **SSD** have a difference in coherence distribution.

Now, let us evaluate coherence distribution of **UD** and **SSD** by using measure of coherence. Because Alice’s two pure states are non-orthogonal, the initial ensemble consists of nonzero coherence. Now, we investigate how the unitary operator $U_{AB}$ distributes the coherence of the initial ensemble. First, let us consider the maximum RQC of system *B*. The maximum RQC of system *B* is expressed, as [15,33]

(9) $$\begin{array}{c} C_{\mathrm{rel}.\mathrm{ent}}^{\left(⋆\right)}\left(\rho_{B}\right)=1-H\left(4q_{1}q_{2}\left\{1-\left(1-\alpha_{1}\right)\left(1-\alpha_{2}\right)\right\}\right). \end{array}$$

Hence, $C_{\mathrm{rel}.\mathrm{ent}}^{\left(⋆\right)}\left(\rho_{B}\right)$ is expressed as a function of $\left(\alpha_{1},\alpha_{2}\right)$. Here, H(x) is a function of entropy: $H\left(x\right)=-\sum_{k=0}^{1}\frac{1+{\left(-1\right)}^{k}\sqrt{1-x}}{2}\log_{2}\frac{1+{\left(-1\right)}^{k}\sqrt{1-x}}{2}$, where x∈[0,1]. According to Equation (9), as $\left(1-\alpha_{1}\right)\left(1-\alpha_{2}\right)$ becomes closer to $s^{2}$, $C_{\mathrm{rel}.\mathrm{ent}}^{\left(⋆\right)}\left(\rho_{B}\right)$ becomes closer to the maximum. This implies that if Bob performs an optimal unambiguous discrimination, then $C_{\mathrm{rel}.\mathrm{ent}}^{\left(⋆\right)}\left(\rho_{B}\right)$ will reach the maximum.

Furthermore, when $\left(\alpha_{1},\alpha_{2}\right)$ corresponds to the optimal measurement that exists on curve $Q_{1}PQ_{2}$, two post-measurement states $|\phi_{1}\rangle$ and $|\phi_{2}\rangle$ overlap completely [14,16]. This implies that, in **UD**, the coherence in Alice’s initial ensemble is localized to Bob’s auxiliary system via unitary operator $U_{AB}$.

In this time, let us assume that Bob and Charlie perform sequential state discrimination. If vector $\left(\alpha_{1},\alpha_{2}\right)$ that correponds to Bob’s measurement is on curve $Q_{1}PQ_{2}$, then Charlie cannot perform unambiguous discrimination. Therefore, the vector $\left(\alpha_{1},\alpha_{2}\right)$ should be inside sector $Q_{1}OQ_{2}$. Because the success probability of sequential state discrimination is given as function $P_{s}^{\left(B,C\right)}\left(\alpha_{1},\alpha_{2}\right)$, the optimal vector $\left(\alpha_{1},\alpha_{2}\right)$ always satisfies the equality $\vec{\nabla }P_{s}^{\left(B,C\right)}\left(\alpha_{1},\alpha_{2}\right)=0$. We can numerically verify that a vector, satisfying a zero-gradient condition, will always be on curve $B_{1}A_{1}A_{2}B_{2}$. Every vector on this curve satisfies:

(10) $$\begin{array}{c} \left(1-\alpha_{1}\right)\left(1-\alpha_{2}\right)=s\left(={\left(\sqrt{s}\right)}^{2}\right). \end{array}$$

Equation (10) is equivalent to an argument proposed by J. A. Bergou et al. [10].

Next, we investigate symmetrized discord to understand the RQC localization in **SSD**. Here, entropic quantum discord is derived as [11,15]

(11) $$\begin{array}{ccc} D_{A}\left(\rho_{AB}\right) & = & H\left(\tau_{A}\right)-H\left(\tau_{E}\right)+H\left(\tau_{B}-\tau_{ABE}\right),\\ D_{B}\left(\rho_{AB}\right) & = & H\left(\tau_{B}\right)-H\left(\tau_{E}\right)+H\left(\tau_{A}-\tau_{ABE}\right). \end{array}$$

Here, the tangles $\tau_{A},\tau_{B},\tau_{E},\tau_{ABE}$ are given as

(12) $$\begin{array}{ccc} \tau_{A} & = & 4q_{1}q_{2}\{1-\frac{s^{2}}{\left(1-\alpha_{1}\right)\left(1-\alpha_{2}\right)}\},\\ \tau_{B} & = & 4q_{1}q_{2}\left\{1-\left(1-\alpha_{1}\right)\left(1-\alpha_{2}\right)\right\},\\ \tau_{E} & = & 4q_{1}q_{2}\left(1-s^{2}\right),\\ \tau_{ABE} & = & 4q_{1}q_{2}\{1-\frac{s^{2}}{\left(1-\alpha_{1}\right)\left(1-\alpha_{2}\right)}\}\left\{1-\left(1-\alpha_{1}\right)\left(1-\alpha_{2}\right)\right\}. \end{array}$$

Because $\rho_{AB}$ is rank-2, Equation (11) is derived from the Koashi–Winter formula [48].

According to Equation (11) and Equation (12), symmetrized discord is also given as a function of $\left(\alpha_{1},\alpha_{2}\right)$. Therefore, we numerically find an optimal vector that maximizes the symmetrized discord. Consequently, we can see that the optimal vector $\left(\alpha_{1},\alpha_{2}\right)$ always exists on a curve $A_{1}B_{1}B_{2}A_{2}$. This implies that, in **SSD**, the coherence in Alice’s initial ensemble is distributed to both system *A* and *B*. In other words, the coherence of $\rho_{AB}$ is localized to neither system *A* nor *B*.

**Observation** **1.** *Suppose that Alice prepares one of two pure states. In **UD**, the unitary operator $U_{AB}$ localizes the coherence in Alice’s initial ensemble into system B. Meanwhile, in **SSD**, $U_{AB}$ distributes the coherence in Alice’s initial ensemble to both systems A and B.*

From Observation 1, we propose that coherence distribution obviously depends on which strategy Bob chooses. If Bob chooses **UD**, the coherence in the initial ensemble is localized in the ancilla system. If Bob chooses **SSD**, the coherence in the initial ensemble is not localized in *B*.

### 4.2. Coherence Distribution in **AOSD1** and **AOSD2**

Here, we investigate the coherence distribution in the unitary operator $V_{AB}$, where assisted optimal state discrimination is performed.

The unitary operator $V_{AB}$ transforms Alice’s initial ensemble into the following:

$$\begin{array}{c} \omega_{AB}=V_{AB}\left\{{\left(q_{1}|\psi_{1}\rangle \langle \psi_{1}|+q_{2}|\psi_{2}\rangle \langle \psi_{2}|\right)}_{A}⊗|b\rangle {\langle b|}_{B}\right\}V_{AB}^{†}. \end{array}$$

According to Zhang, Chen, Kwek, and Vedral [22], zero concurrence in the average state $\sigma_{AB}$ is analytically expressed as

$$\begin{array}{c} \delta =-\theta ,\;\;\beta =\tan^{-1}\left[\frac{q_{1}\left|\alpha_{1}\right|\sqrt{1-|\alpha_{1}{|}^{2}}}{q_{2}\left|\alpha_{2}\right|\sqrt{1-|\alpha_{2}{|}^{2}}}\right]. \end{array}$$

Because the success probability is not affected by the two parameters δ and θ, we can assume that δ=θ=0 without any loss of generality. δ and θ do not affect the success probability. In other words, $\langle \psi_{1}|\psi_{2}\rangle$ can be considered as a real number.

Assume that $q_{1}\leq q_{2}$ holds. When $\langle \psi_{1}|\psi_{2}\rangle \leq \sqrt{q_{1}/q_{2}}$, Bob’s optimal strategy is described as **AOSD2**. In this case, $\rho_{AB}$ is given, as follows [22]:

$$\begin{array}{c} \omega_{AB}=|\xi_{1}\rangle {\langle \xi_{1}|}_{A}⊗|0\rangle {\langle 0|}_{B}+|\Phi \rangle {\langle \Phi |}_{A}⊗|\xi_{2}\rangle {\langle \xi_{2}|}_{B}. \end{array}$$

Here, $|\xi_{i}\rangle$ is provided, as follows:

$$\begin{array}{ccc} |\xi_{1}\rangle & = & \frac{\sqrt{q_{1}q_{2}}}{\sqrt{q_{1}|\alpha_{1}{|}^{2}+q_{1}{\left|\alpha_{2}\right|}^{2}}}\left(\sqrt{1-|\alpha_{1}{|}^{2}}\alpha_{2}|0\rangle -\sqrt{1-|\alpha_{2}{|}^{2}}\alpha_{1}|1\rangle \right),\\ |\xi_{2}\rangle & = & \frac{\sqrt{\left(1-\right|\alpha_{1}{|}^{2})|\alpha_{1}{|}^{2}p_{1}^{2}+(1-|\alpha_{2}{|}^{2})|\alpha_{2}{|}^{2}p_{2}^{2}}}{\sqrt{q_{1}|\alpha_{1}{|}^{2}+q_{1}{\left|\alpha_{2}\right|}^{2}}}|0\rangle +\sqrt{q_{1}|\alpha_{1}{|}^{2}+q_{1}{\left|\alpha_{2}\right|}^{2}}\frac{\alpha_{1}}{|\alpha_{1}|}|1\rangle . \end{array}$$

According to Ref. [22], $D_{B}\left(\omega_{AB}\right)$ is nonzero. This implies that the RQC in $\omega_{AB}$ is not localized in system *B*. According to structure of $\omega_{AB}$, the partial state $\omega_{A}$ is an ensemble that is composed of two nonorthogonal pure states. Hence, RQC is not localized in system *B*.

Meanwhile, if $\sqrt{q_{1}/q_{2}}\leq \langle \psi_{1}|\psi_{2}\rangle \leq 1$, Bob’s optimal strategy is described as **AOSD1**. In this case, $\omega_{AB}$ is given, as follows [22]:

$$\begin{array}{c} \omega_{AB}=|1\rangle {\langle 1|}_{A}⊗{\left\{p_{1}|1\rangle \langle 1|+p_{2}|\mu \rangle \langle \mu |\right\}}_{B}. \end{array}$$

Here, |μ〉 becomes

$$\begin{array}{c} |\mu \rangle =\sqrt{1-s^{2}}|0\rangle +\alpha_{2}e^{i\delta }|1\rangle . \end{array}$$

Clearly, $\rho_{A}$ is an incoherent state. Furthermore, $D_{B}\left(\omega_{AB}\right)$ is obviously zero, since $\omega_{AB}$ is a product state. This implies that the RQC in $\omega_{AB}$ has the potential to be localized in system *B*. According to Zhang, Chen, Kwek, and Vedral [22], the partial state $\omega_{A}$ is given as a pure incoherent state. Therefore, the RQC shown in $\omega_{AB}$ is obviously localized in system *B*.

**Observation** **2.** *Suppose that Alice prepares one of two pure states. In **AOSD2**, the coherence in $\omega_{AB}$ is not localized in system B. Meanwhile, in **AOSD1**, the coherence in $\omega_{AB}$ is localized in system B.*

From Observation 2, we can tell that the coherence distribution in **AOSD** also depends on which strategy Bob chooses. If Bob chooses **AOSD2**, the coherence is distributed to both system *A* and *B*. If Bob chooses **AOSD1**, the coherence is localized in system *B*. These results imply that the structure of generalized measurements performing unambiguous discrimination, sequential state discrimination, and assisted optimal state discrimination can be understood by coherence distribution.

**Figure 6:**
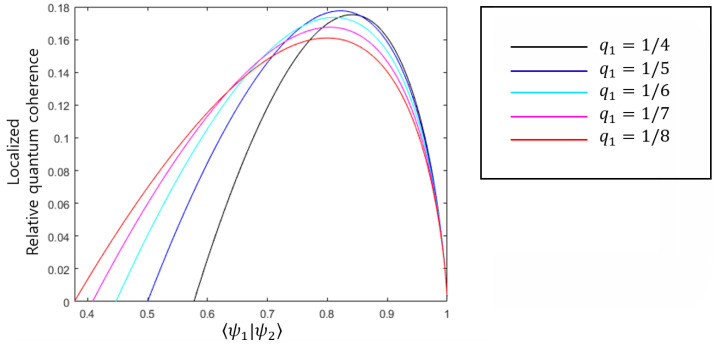
RQC of system *B* in **AOSD**. The black, dark-blue, light-blue, purple and red solid lines correspond to $q_{1}=1/4,1/5,1/6,1/7$, and 1/8, respectively. This figure implies that RQC is nonzero when inner product $\langle \psi_{1}|\psi_{2}\rangle$ satisfies $\sqrt{q_{1}/q_{2}}\leq \langle \psi_{1}|\psi_{2}\rangle \leq 1$. In this region, the optimal strategy of Bob is to discriminate only $|\psi_{2}\rangle$ out of two pure states of Alice. In this case, **AOSD** is equivalent to **AOSD1**.

Zhang, Chen, Kwek, and Vedral [22] attempted to explain the structure of **AOSD** in terms of quantum discord (or dissonance) However, if there is significant overlap between two pure states, the entropic quantum discord becomes zero. In other words, the relationship between **AOSD** and entropic quantum discord holds only when specific constraints concerning overlap are imposed. Meanwhile, According to Figure 6, the RQC of system *B* is nonzero in the region $\sqrt{q_{1}/q_{2}}\leq \langle \psi_{1}|\psi_{2}\rangle \leq 1$, where it is assumed that $q_{1}<q_{2}$. Therefore, Observation 2 implies that there is a consistent relationship between coherence distribution and **AOSD**.

## 5. Generalization of Coherence Distribution to 2 Mixed States Discrimination

Here, let us suppose that Alice prepares a mixed state $\rho_{i}\in \left\{\rho_{1},\rho_{2}\right\}$, with a prior probability $q_{i}$. If the support of $\rho_{1}$ and $\rho_{2}$ do not ovelap completely, then Bob’s measurement can discriminate Alice’s mixed state without any error [49,50]. Additionally, the POVM element $M_{1}$($M_{2}$) consists of the kernel of $\rho_{2}$($\rho_{1}$). Unfortunately, the general structure of POVM, which can discriminate a general mixed state without an error, has been unknown yet. However, if Alice’s Hilbert space has a special form in $H_{A}=H_{A}^{\left(1\right)}⊕H_{A}^{\left(2\right)}⊕\cdots ⊕H_{A}^{\left(R\right)}$, the structure of POVM for unambiguous discrimination is well known [51]. If we assume that the state space $S(H_{A})$ is constructed from this form of Hilbert space, then Alice’s mixed state can be expressed as

(13) $$\begin{array}{c} \rho_{i}=r_{i}^{\left(1\right)}|r_{i}^{\left(1\right)}\rangle \langle r_{i}^{\left(1\right)}|⊕\cdots ⊕r_{i}^{\left(R\right)}|r_{i}^{\left(R\right)}\rangle \langle r_{i}^{\left(R\right)}|. \end{array}$$

Here, *R* is maximum rank of $\rho_{i}$. If $|\langle r_{1}^{\left(Q\right)}|r_{2}^{\left(Q\right)}\rangle |<1$ holds for all of Q∈{1,2,⋯,R}, then Bob can discriminate Alice’s mixed states without any error. Fortunately, Bob’s POVM consists of *R* sub-POVMs, where each sub-POVM discriminates $|r_{i}^{\left(Q\right)}\rangle ,|r_{i}^{\left(Q\right)}\rangle \in H_{A}^{\left(Q\right)}$(Q∈{1,2,⋯,R}) without any error. This implies that we can consistently substitute our argument of pure states into that of mixed states (the detailed evaluation can be found in Appendix A and Appendix B).

### 5.1. Generalized Witness of RQC Localization

Now, let us consider $\rho_{i}\in S\left(H_{A}^{\left(1\right)}⊕\cdots ⊕H_{A}^{\left(R\right)}\right)$. That is, unitary operator $U_{AB}\in \left\{U_{AB},V_{AB}\right\}$ maps $S(H_{A}^{\left(1\right)}⊕\cdots ⊕H_{A}^{\left(R\right)}⊗H_{B})$ onto itself. This unitary operator $U_{AB}$ transforms Alice’s initial ensemble into an average state expressed as

(14) $$\begin{array}{ccc} \rho_{AB} & = & U_{AB}\left\{{\left(q_{1}\rho_{1}+q_{2}\rho_{2}\right)}_{A}⊗|b\rangle {\langle b|}_{B}\right\}U_{AB}^{†}=\overset{R}{\underset{Q=1}{⨁}}\sigma_{AB}^{\left(Q\right)}=\overset{R}{\underset{Q=1}{⨁}}\left(\mathrm{Tr}\sigma_{AB}^{\left(Q\right)}\right){\sigma^{\prime }}_{AB}^{\left(Q\right)}. \end{array}$$

Here, ${\sigma^{\prime }}_{AB}^{\left(Q\right)}=\sigma_{AB}^{\left(Q\right)}/\mathrm{Tr}\sigma_{AB}^{\left(Q\right)}\in S\left(H_{A}^{\left(Q\right)}⊗H_{B}\right)$ is a normalized positive-semidefinite operator. Because every ${\sigma^{\prime }}_{AB}^{\left(Q\right)}$ is of rank-2, then the Koashi–Winter formula can be used in order to obtain a lower bound for the entropic quantum discord and symmetrized discord:

$$\begin{array}{ccc} D_{A}\left(\rho_{AB}\right) & \geq & \sum_{Q=1}^{R}\left(\mathrm{Tr}\sigma_{AB}^{\left(Q\right)}\right)D_{A}\left({\sigma^{\prime }}_{AB}^{\left(Q\right)}\right)\equiv W_{A}\left(\rho_{AB}\right),\\ D_{B}\left(\rho_{AB}\right) & \geq & \sum_{Q=1}^{R}\left(\mathrm{Tr}\sigma_{AB}^{\left(Q\right)}\right)D_{B}\left({\sigma^{\prime }}_{AB}^{\left(Q\right)}\right)\equiv W_{A}\left(\rho_{AB}\right),\\ D_{AB}\left(\rho_{AB}\right) & \geq & \sum_{Q=1}^{R}\left(\mathrm{Tr}\sigma_{AB}^{\left(Q\right)}\right)D_{AB}\left({\sigma^{\prime }}_{AB}^{\left(Q\right)}\right)\equiv W_{AB}\left(\rho_{AB}\right). \end{array}$$

Because $\delta_{X}\left({\sigma^{\prime }}_{AB}^{\left(Q\right)}\right)\geq D_{X}\left({\sigma^{\prime }}_{AB}^{\left(Q\right)}\right)$ holds for all of Q∈{1,2,⋯,R} and X∈{A,B}, the lower bound of the RQC discrepancy in $\rho_{AB}$ becomes:

(15) $$\begin{array}{ccc} \delta_{A}\left(\rho_{AB}\right)=\sum_{Q=1}^{R}\left(\mathrm{Tr}\sigma_{AB}^{\left(Q\right)}\right)\delta_{A}\left({\sigma^{\prime }}_{AB}^{\left(Q\right)}\right) & \geq & W_{A}\left(\rho_{AB}\right),\\ \delta_{B}\left(\rho_{AB}\right)=\sum_{Q=1}^{R}\left(\mathrm{Tr}\sigma_{AB}^{\left(Q\right)}\right)\delta_{B}\left({\sigma^{\prime }}_{AB}^{\left(Q\right)}\right) & \geq & W_{B}\left(\rho_{AB}\right),\\ \sqrt{\delta_{A}\left(\rho_{AB}\right)\delta_{B}\left(\rho_{AB}\right)} & \geq & W_{AB}\left(\rho_{AB}\right). \end{array}$$

In Equation (15), $W_{A}\left(\rho_{AB}\right),W_{B}\left(\rho_{AB}\right)$, and $W_{AB}\left(\rho_{AB}\right)$ describe the generalized RQC discrepancy in $\rho_{AB}$. If R=1, the generalized RQC discrepancy is equivalent to the entropic quantum discord and symmetrized discord of pure states. Equation (15) implies that the generalized RQC discrepancy can be a witness of RQC localization.

### 5.2. Generalization of Coherence Distribution in **UD** and **SSD**

In cases of **UD** and **SSD**, unitary operator $U_{AB}$ has the specific form of $U_{AB}=U_{AB}^{\left(1\right)}⊕\cdots ⊕U_{AB}^{\left(R\right)}$. Here, $U_{AB}^{\left(Q\right)}$ is sub-unitary and it is defined as

$$\begin{array}{ccc} & & U_{AB}^{\left(Q\right)}{|r_{i}^{\left(Q\right)}\rangle }_{A}⊗{|b\rangle }_{B}={|s_{i}^{\left(Q\right)}\rangle }_{A}⊗\left\{\sqrt{\alpha_{i}^{\left(Q\right)}}{|i\rangle }_{B}+\sqrt{1-\alpha_{i}^{\left(Q\right)}}{|0\rangle }_{B}\right\}. \end{array}$$

After $U_{AB}$ is terminated, then Bob’s local projective measurement ${\left\{|i\rangle \langle i|\right\}}_{i=0}^{2}$ is performed on system *B*.

The necessary and sufficient condition that $U_{AB}^{\left(Q\right)}$ must exist is expressed in a similar manner while using Equation (10). This implies that Bob’s measurement consists of *R* real vectors $\left(\alpha_{1}^{\left(Q\right)},\alpha_{2}^{\left(Q\right)}\right)\in C^{\left(Q\right)}$. Here, each convex set $C^{\left(Q\right)}$ has the same structure as that seen in Figure 5. Therefore, we can apply our argument of **SSD** to cases, including mixed states [14].

From the structure of Equation (14), the maximum RQC of $\rho_{AB}$ is explicitly derived as

$$\begin{array}{ccc} C_{\mathrm{rel}.\mathrm{ent}}^{\left(⋆\right)}\left(\rho_{B}\right) & = & \sum_{Q=1}^{R}\left(\mathrm{Tr}\sigma_{AB}^{\left(Q\right)}\right)\left\{1-H\left(\tau_{B}^{\left(Q\right)}\right)\right\}=1-\sum_{Q=1}^{R}\left(\mathrm{Tr}\sigma_{AB}^{\left(Q\right)}\right)H\left(\tau_{B}^{\left(Q\right)}\right). \end{array}$$

If the prior probabilities of the two mixed states $\rho_{1}$ and $\rho_{2}$ are given as $q_{1}$ and $q_{2}$, respectively, then the entire unambiguous discrimination consists of *R* discrimination problems, where the pure state $|r_{i}^{\left(Q\right)}\rangle \in \{|r_{1}^{\left(Q\right)}\rangle ,|r_{2}^{\left(Q\right)}\rangle \}$ is prepared with the prior probability $q_{i}r_{i}^{\left(Q\right)}/\mathrm{Tr}\left[\sigma_{AB}^{\left(Q\right)}\right]$. Hence, tangle $\tau_{B}^{\left(Q\right)}$ is given as

$$\begin{array}{c} \tau_{B}^{\left(Q\right)}=4\frac{q_{1}r_{1}^{\left(Q\right)}}{\mathrm{Tr}\sigma_{AB}^{\left(Q\right)}}\frac{q_{2}r_{2}^{\left(Q\right)}}{\mathrm{Tr}\sigma_{AB}^{\left(Q\right)}}\left\{1-\left(1-\alpha_{1}^{\left(Q\right)}\right)\left(1-\alpha_{2}^{\left(Q\right)}\right)\right\}. \end{array}$$

For all of Q∈{1,2,⋯,R}, if $\left(\alpha_{1}^{\left(Q\right)},\alpha_{2}^{\left(Q\right)}\right)$ is on the boundary of the convex set $C^{\left(Q\right)}$, the maximum RQC of $\rho_{B}$ becomes maximized. Meanwhile, the support between the two post-measurement states is completely overlapped. Therefore, system *A* does not contain coherence. In conclusion, we can successfully extend Observation 1 to cases of mixed states.

**Observation** **3.** *Suppose that Alice prepares one out of two mixed state. In **UD**, the unitary operator $U_{AB}$ localizes the coherence in Alice’s initial ensemble into system B. In **SSD**, $U_{AB}$ distributes the coherence in Alice’s initial ensemble to both system A and B.*

### 5.3. Generalization of Coherence Distribution in **AOSD1** and **AOSD2**

Likewise, the unitary operator $V_{AB}$ also has a specific form of $V_{AB}=V_{AB}^{\left(1\right)}⊕\cdots ⊕V_{AB}^{\left(R\right)}$. Here, $V_{AB}^{\left(Q\right)}$ performs

$$\begin{array}{ccc} & & V_{AB}^{\left(Q\right)}{|r_{1}^{\left(Q\right)}\rangle }_{A}⊗{|0\rangle }_{B}=\sqrt{1-|\alpha_{1}^{\left(Q\right)}{|}^{2}}{|0^{\left(Q\right)}\rangle }_{A}⊗{|0\rangle }_{B}+\alpha_{1}^{\left(Q\right)}{|\phi^{\left(Q\right)}\rangle }_{A}⊗{|1\rangle }_{B},\\ & & V_{AB}^{\left(Q\right)}{|r_{2}^{\left(Q\right)}\rangle }_{A}⊗{|0\rangle }_{B}=\sqrt{1-|\alpha_{2}^{\left(Q\right)}{|}^{2}}{|1^{\left(Q\right)}\rangle }_{A}⊗{|0\rangle }_{B}+\alpha_{1}^{\left(Q\right)}{|\phi^{\left(Q\right)}\rangle }_{A}⊗{|1\rangle }_{B}. \end{array}$$

Here, $\left\{|0^{\left(Q\right)}\rangle ,|1^{\left(Q\right)}\rangle \right\}$ is an orthonormal basis consisting of $H_{A}^{\left(Q\right)}$. Moreover, $|\phi^{\left(Q\right)}\rangle =\cos\beta_{Q}|0^{\left(Q\right)}\rangle +\sin\beta_{Q}e^{i\delta_{Q}}|1^{\left(Q\right)}\rangle$. The inner product between two vectors $\langle r_{1}^{\left(Q\right)}|r_{2}^{\left(Q\right)}\rangle$ is expressed as $\alpha_{1}^{\left(Q\right)}\alpha_{2}^{\left(Q\right)}=\left|\langle r_{1}^{\left(Q\right)}|r_{2}^{\left(Q\right)}\rangle \right|e^{i\delta_{Q}}$. After $V_{AB}$ is terminated, Bob performs the projective measurement {|0〉〈0|,|1〉〈1|} on his system.

Likewise, in **UD** and **SSD**, in **AOSD**, $V_{AB}$ maps $S(H_{A}^{\left(1\right)}⊕\cdots ⊕H_{A}^{\left(R\right)}⊗H_{B})$ to itself.

Therefore, we can successfully extend Observation 2 to instances of mixed states.

**Observation** **4.** *Suppose that Alice prepares one out of two mixed states. Subsequently, In **AOS12**, the coherence of an initial bipartite ensemble is localized in system B. Meanwhile, in an **AOSD2**, the coherence of in an initial bipartite ensemble is not localized in system B.*

## 6. Conclusions and Future Work

**Figure 7:**
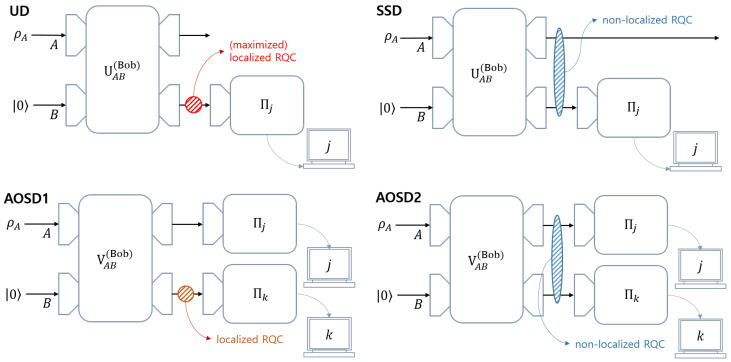
The coherence distribution in **UD**, **SSD**, **AOSD1**, and **AOSD2**. In **UD**, the unitary operator localizes the coherence in Alice’s initial ensemble ($\rho_{A}$) into Bob’s auxiliary system. In **SSD**, the unitary operator distributes the coherence in Alice’s initial ensemble into both Alice and Bob’s systems. In **AOSD1**, the unitary operator localizes the coherence in Alice’s initial ensemble into Bob’s system. In **AOSD2**, the unitary operator distributes the coherence in Alice’s initial ensemble to both Alice’s and Bob’s systems. This figure shows that the way where coherence distribution occurs depends on the structure of Bob’s measurement.

In this paper, we investigated how the distribution of coherence occurs when generalized measurement performs various types of unambiguous discrimination. By investigating the RQC localization, we showed that the coherence distribution depends on the types of quantum unambiguous discrimination (See Figure 7). In other words, we found that the types of quantum unambiguous discrimination determine how coherence is distributed in performing unambiguous discrimination. Further, we showed that our argument could be extended to cases, including two mixed states.

In fact, generalized measurement can be applied to performing not only unambiguous discrimination, but also minimum error discrimination [52,53,54,55,56,57,58] and the fixed rate of inconclusive result [59,60]. Therefore, while using the argument provided in this work, it is interesting to investigate whether coherence distribution can explain the structure of generalized measurements performing other quantum state discriminations. In other words, it can be important to study coherence distribution in various quantum state discrimination strategies.

Beyond the discrimination tasks, a generalized measurement can be applied to various quantum information and computation tasks. The quantum classifier [32], whose efficiency is given as $O(\sqrt{N})$ for the number of data *N*, can be a good example. In training a quantum classifier, which is composed of (a), a parametric multilayer quantum circuit performed on both feature register and index register (b) local projective measurements performed on index register, it is shown that the successfully trained parametric multilayer quantum circuit distributes coherence in the index register [61]. It can be understood that, in the generalized measurement of the problem, the measurement on the index register is important. Further, in the quantum classifier, the Grover algorithm depletes the index register’s coherence as a resource [28,30]. Therefore, investigating coherence distribution in generalized measurement may provide a clue for quantum advantage in quantum tasks.

## Appendix A. Deriving the Relationship between Discord and Generalized Witness in the Case of Two Mixed States

Firstly, the rank-2 state is considered to be

(A1) $$\begin{array}{ccc} \rho_{AB} & = & q_{1}r_{1}|s_{1}\rangle {\langle s_{1}|}_{A}⊗|B_{1}\rangle {\langle B_{1}|}_{B}⊕q_{2}r_{2}|s_{2}\rangle {\langle s_{2}|}_{A}⊗|B_{2}\rangle {\langle B_{2}|}_{B}\\ & + & q_{1}{\bar{r}}_{1}|{\bar{s}}_{1}\rangle {\langle {\bar{s}}_{1}|}_{A}⊗|{\bar{B}}_{1}\rangle {\langle {\bar{B}}_{1}|}_{B}⊕q_{2}{\bar{r}}_{2}|{\bar{s}}_{2}\rangle {\langle {\bar{s}}_{2}|}_{A}⊗|{\bar{B}}_{2}\rangle {\langle {\bar{B}}_{2}|}_{B}\\ & = & \sigma_{AB}⊕{\bar{\sigma }}_{AB}. \end{array}$$

Although we consider the case in which the states are rank-2, our method can be extended to the rank-*N* states case. Obviously, the bipartite state $\rho_{AB}$ resides in state space $S(H_{AB}⊕{\bar{H}}_{AB})$. Here, let us consider the entropic quantum discord $D_{A}\left(\rho_{AB}\right)$. Since Equation (A1) is decomposed as a direct sum between the two rank-2 positive semidefinite operator, POVM ${\left\{M_{k}\right\}}_{k=1}^{N}$ can also be expressed as

(A2) $$\begin{array}{c} M_{k}^{\left(A\right)}=[\begin{array}{cc} \Pi_{k} & \Gamma_{k}\\ \Gamma_{k}^{†} & {\bar{\Pi }}_{k} \end{array}]. \end{array}$$

Here, $\Pi_{k}$(${\bar{\Pi }}_{k}$) is a positive semidefinite operator defined over Hilbert space H($\bar{H}$) and $\Gamma_{k}$(${\bar{\Gamma }}_{k}$) is a linear map in which $H\to \bar{H}$($\bar{H}\to H$).

As $M_{k}$ is positive semidefinite, $\langle v|M_{k}|v\rangle$ is also positive semidefinite for all $|v\rangle \in H⊕\bar{H}$. Obviously, $|x\rangle ⊕|\bar{0}\rangle$ and $|0\rangle ⊕|\bar{x}\rangle$ are also elements of $H⊕\bar{H}$. Here, |0〉($|\bar{0}\rangle$) is zero vector in sub Hilbert space H($\bar{H}$). Thus, $\left(\langle x|⊕\langle \bar{0}|\right)M_{k}\left(|x\rangle ⊕|\bar{0}\rangle \right)=\langle x|\Pi_{k}|x\rangle$ and $\left(\langle 0|⊕\langle \bar{x}|\right)M_{k}\left(|0\rangle ⊕|\bar{x}\rangle \right)=\langle \bar{x}|{\bar{\Pi }}_{k}|\bar{x}\rangle$ are also positive semidefinite for all |x〉∈H and $|\bar{x}\rangle \in \bar{H}$. In other words, if $M_{k}$ is positive semidefinite, then $\Pi_{k}$ and ${\bar{\Pi }}_{k}$ are also positive semidefinite.

The completeness of two sub-POVMs ${\left\{\Pi_{k}\right\}}_{k=1}^{N},{\left\{{\bar{\Pi }}_{k}\right\}}_{k=1}^{N}$ is directly checked as

$$\begin{array}{c} \sum_{k=1}^{N}M_{k}^{\left(A\right)}=\sum_{k=1}^{N}[\begin{array}{cc} \Pi_{k} & \Gamma_{k}\\ \Gamma_{k}^{†} & {\bar{\Pi }}_{k} \end{array}]=[\begin{array}{cc} \sum_{k=1}^{N}\Pi_{k} & \sum_{k=1}^{N}\Gamma_{k}\\ \sum_{k=1}^{N}\Gamma_{k}^{†} & \sum_{k=1}^{N}{\bar{\Pi }}_{k} \end{array}]=[\begin{array}{cc} I_{A} & O\\ O & {\bar{I}}_{A} \end{array}]=I_{A}. \end{array}$$

Here, O is the null operator. $I_{A}$ is an identity operator over Hilbert space $H_{AB}$. $I_{A}$(${\bar{I}}_{A}$) is an identity operator over Hilbert space $H_{A}$(${\bar{H}}_{A}$). Therefore, if $\sum_{k}M_{k}=I_{A}$, then the equalities $\sum_{k}\Pi_{k}=I_{A},\sum_{k}{\bar{\Pi }}_{k}={\bar{I}}_{A},\sum_{k}\Gamma_{k}=O$ automatically hold. Without loss of generality, we can assume that every nonzero operator $\Gamma_{k}$ satisfies $\sum_{k}\Gamma_{k}=O$. That is, because the explicit form of $\Gamma_{k}$ does not affect the expression for the lower bound of quantum discord. Therefore, two sub-POVMs ${\left\{\Pi_{k}\right\}}_{k=1}^{N}$ and ${\left\{{\bar{\Pi }}_{k}\right\}}_{k=1}^{N}$ also satisfy completeness condition.

Substituting Equation (A2) into Equation (A1), we obtain the expression for $p_{k}$ and $\rho_{B|k}$:

$$\begin{array}{ccc} p_{k} & = & \mathrm{Tr}\left(\sigma_{AB}\Pi_{k}⊗I_{B}\right)+\mathrm{Tr}\left({\bar{\sigma }}_{AB}{\bar{\Pi }}_{k}⊗I_{B}\right),\\ \rho_{B|k} & = & \frac{{\mathrm{Tr}}_{A}\left(\sigma_{AB}\Pi_{k}⊗I_{B}\right)+{\mathrm{Tr}}_{A}\left({\bar{\sigma }}_{AB}{\bar{\Pi }}_{k}⊗I_{B}\right)}{p_{k}}. \end{array}$$

Using concavity of von Neumann entropy, we derive the inequality of conditional entropy as

$$\begin{array}{ccc} & & \sum_{k=1}^{N}p_{k}S\left(\rho_{B|k}\right)=\sum_{k=1}^{N}\left\{\mathrm{Tr}\left(\sigma_{AB}\Pi_{k}⊗I_{B}\right)+\mathrm{Tr}\left({\bar{\sigma }}_{AB}{\bar{\Pi }}_{k}⊗I_{B}\right)\right\}S\left(\frac{{\mathrm{Tr}}_{A}\left(\sigma_{AB}\Pi_{k}⊗I_{B}\right)+{\mathrm{Tr}}_{A}\left({\bar{\sigma }}_{AB}{\bar{\Pi }}_{k}⊗I_{B}\right)}{\mathrm{Tr}\left(\sigma_{AB}\Pi_{k}⊗I_{B}\right)+\mathrm{Tr}\left({\bar{\sigma }}_{AB}{\bar{\Pi }}_{k}⊗I_{B}\right)}\right)\\ & & \geq \sum_{k=1}^{N}\left\{\mathrm{Tr}\left(\sigma_{AB}\Pi_{k}⊗I_{B}\right)S\left(\frac{{\mathrm{Tr}}_{A}\left(\sigma_{AB}\Pi_{k}⊗I_{B}\right)}{\mathrm{Tr}(\sigma_{AB}\Pi_{k}⊗I_{B})}\right)+\mathrm{Tr}\left({\bar{\sigma }}_{AB}{\bar{\Pi }}_{k}⊗I_{B}\right)S\left(\frac{{\mathrm{Tr}}_{A}\left({\bar{\sigma }}_{AB}{\bar{\Pi }}_{k}⊗I_{B}\right)}{\mathrm{Tr}({\bar{\sigma }}_{AB}{\bar{\Pi }}_{k}⊗I_{B})}\right)\right\}\\ & & =\left(\mathrm{Tr}\sigma_{AB}\right)\sum_{k=1}^{N}p_{k}^{\prime }S\left({\sigma^{\prime }}_{B|k}\right)+\left(\mathrm{Tr}{\bar{\sigma }}_{AB}\right)\sum_{k=1}^{N}{\bar{p}}_{k}^{\prime }S\left({{\bar{\sigma }}^{\prime }}_{B|k}\right). \end{array}$$

Here, we define $\sigma_{AB}^{\prime }$ and ${{\bar{\sigma }}^{\prime }}_{AB}$ as $\sigma_{AB}^{\prime }:=\sigma_{AB}/\mathrm{Tr}\left[\sigma_{AB}\right]$ and ${\bar{\sigma }}_{AB}^{\prime }$:=${\bar{\sigma }}_{AB}/\mathrm{Tr}\left[{\bar{\sigma }}_{AB}\right]$.

In order that the quantum discord is evaluated, we minimize the conditional entropy under every POVM ${\left\{M_{k}^{\left(A\right)}\right\}}_{k=1}^{N}$ [34]. Since POVM element $M_{k}^{\left(A\right)}$ consists of $\Pi_{k},\Gamma_{k}$ and ${\bar{\Pi }}_{k}$, the minimum conditional entropy satisfies

$$\begin{array}{ccc} & \min & {}_{{\left\{M_{k}^{\left(A\right)}\right\}}_{k=1}^{N}}\sum_{k=1}^{N}p_{k}S\left(\rho_{B|k}\right)=\min_{{\left\{\Pi_{k}\right\}}_{k=1}^{N},{\left\{\Gamma_{k}\right\}}_{k=1}^{N},{\left\{{\bar{\Pi }}_{k}\right\}}_{k=1}^{N}}\sum_{k=1}^{N}p_{k}S\left(\rho_{B|k}\right)\\ & = & \min_{{\left\{\Pi_{k}\right\}}_{k=1}^{N},{\left\{{\bar{\Pi }}_{k}\right\}}_{k=1}^{N}}\sum_{k=1}^{N}p_{k}S\left(\rho_{B|k}\right)\geq \min_{{\left\{\Pi_{k}\right\}}_{k=1}^{N}}\left(\mathrm{Tr}\sigma_{AB}\right)\sum_{k=1}^{N}p_{k}^{\prime }S\left(\sigma_{B|k}^{\prime }\right)\\ & + & \min_{{\left\{{\bar{\Pi }}_{k}\right\}}_{k=1}^{N}}\left(\mathrm{Tr}{\bar{\sigma }}_{AB}\right)\sum_{k=1}^{N}p_{k}^{\prime }S\left({\bar{\sigma }}_{B|k}^{\prime }\right). \end{array}$$

As $p_{k}$ and $\rho_{B|k}$ are independent on $\Gamma_{k}$, conditional entropy is also independent on ${\left\{\Gamma_{k}\right\}}_{k=1}^{N}$. Therefore, the second equality holds. Inequality can be derived from the convexity of von Neumann entropy. We apply the Koashi-Winter formula [48] to final expression. The purification of $\sigma_{AB}^{\prime }$ and ${{\bar{\sigma }}^{\prime }}_{AB}$ is expressed as

$$\begin{array}{ccc} {|\psi \rangle }_{ABE} & = & \sqrt{\frac{q_{1}r_{1}}{q_{1}r_{1}+q_{2}r_{2}}}{|s_{1}\rangle }_{A}⊗{|B_{1}\rangle }_{B}⊗{|e_{1}\rangle }_{E}+\sqrt{\frac{q_{2}r_{2}}{q_{1}r_{1}+q_{2}r_{2}}}{|s_{2}\rangle }_{A}⊗{|B_{2}\rangle }_{B}⊗{|e_{2}\rangle }_{E},\\ {|\bar{\psi }\rangle }_{AB\bar{E}} & = & \sqrt{\frac{q_{1}{\bar{r}}_{1}}{q_{1}{\bar{r}}_{1}+q_{2}{\bar{r}}_{2}}}{|{\bar{s}}_{1}\rangle }_{A}⊗{|{\bar{B}}_{1}\rangle }_{B}⊗{|{\bar{e}}_{1}\rangle }_{\bar{E}}+\sqrt{\frac{q_{2}{\bar{r}}_{2}}{q_{1}{\bar{r}}_{1}+q_{2}{\bar{r}}_{2}}}{|{\bar{s}}_{2}\rangle }_{A}⊗{|{\bar{B}}_{2}\rangle }_{B}⊗{|{\bar{e}}_{2}\rangle }_{\bar{E}}. \end{array}$$

Here, $\left\{|e_{1}\rangle ,|e_{2}\rangle \right\}$($\left\{|{\bar{e}}_{1}\rangle ,|{\bar{e}}_{2}\rangle \right\}$) is orthonormal basis constructing system *E*(system $\bar{E}$). According to the Koashi-Winter formula, we can derive the inequality as [11,15]

$$\begin{array}{c} \min_{{\left\{M_{k}^{\left(A\right)}\right\}}_{k=1}^{N}}\sum_{k=1}^{N}p_{k}S\left(\rho_{B|k}\right)\geq \left(\mathrm{Tr}\sigma_{AB}\right)E_{f}\left(\sigma_{BE}^{\prime }\right)+\left(\mathrm{Tr}{\bar{\sigma }}_{B\bar{E}}\right)E_{f}\left({{\bar{\sigma }}^{\prime }}_{B\bar{E}}\right), \end{array}$$

where $E_{f}\left(\cdot \right)$ is entanglement of formation [62]. We can easity derive the equality $S\left(\rho_{A}\right)-S\left(\rho_{AB}\right)=\left(\mathrm{Tr}\sigma_{AB}\right)\left\{S\left(\sigma_{A}^{\prime }\right)-S\left(\sigma_{AB}^{\prime }\right)\right\}$$-\left(\mathrm{Tr}{\bar{\sigma }}_{AB}\right)\left\{S\left({\bar{\sigma }}_{A}^{\prime }\right)-S\left({\bar{\sigma }}_{AB}^{\prime }\right)\right\}$. Hence, we obtain the inequality:

$$\begin{array}{ccc} D_{A}\left(\rho_{AB}\right) & = & S\left(\rho_{A}\right)-S\left(\rho_{AB}\right)+\min_{{\left\{M_{k}^{\left(A\right)}\right\}}_{k=1}^{N}}\sum_{k=1}^{N}p_{k}S\left(\rho_{B|k}\right)\\ & \geq & \left(\mathrm{Tr}\sigma_{AB}\right)\left\{S\left(\sigma_{A}^{\prime }\right)-S\left(\sigma_{AB}^{\prime }\right)+E_{f}\left(\sigma_{BE}^{\prime }\right)\right\}+\left(\mathrm{Tr}{\bar{\sigma }}_{AB}\right)\left\{S\left({\bar{\sigma }}_{A}^{\prime }\right)-S\left({\bar{\sigma }}_{AB}^{\prime }\right)+E_{f}\left({\bar{\sigma }}_{BE}^{\prime }\right)\right\}\\ & = & \left(\mathrm{Tr}\sigma_{AB}\right)D_{A}\left(\sigma_{AB}^{\prime }\right)+\left(\mathrm{Tr}{\bar{\sigma }}_{AB}\right)D_{A}\left({\bar{\sigma }}_{AB}^{\prime }\right)=W_{A}\left(\rho_{AB}\right). \end{array}$$

A.2 Deriving the relationship between discord $D_{B}\left(\rho_{AB}\right)$ and generalized witness $W_{B}\left(\rho_{AB}\right)$ Likewise, we can obtain the expression for $p_{k}$ and $\rho_{A|k}$ as

$$\begin{array}{ccc} p_{k} & = & \mathrm{Tr}\left(\sigma_{AB}I_{A}⊗M_{k}^{\left(B\right)}\right)+\mathrm{Tr}\left({\bar{\sigma }}_{AB}{\bar{I}}_{A}⊗M_{k}^{\left(B\right)}\right),\\ \rho_{A|k} & = & \frac{{\mathrm{Tr}}_{B}\left(\sigma_{AB}I_{A}⊗M_{k}^{\left(B\right)}\right)⊕{\mathrm{Tr}}_{B}\left({\bar{\sigma }}_{AB}{\bar{I}}_{A}⊗M_{k}^{\left(B\right)}\right)}{p_{k}}. \end{array}$$

Here, $\rho_{A|k}$ is decomposed as direct sum between the two positive semidefinite operators. Therefore, von Neumann entropy $S(\rho_{A|k})$ satisfies

$$\begin{array}{ccc} & & S\left(\rho_{A|k}\right)=S(\frac{\mathrm{Tr}(\sigma_{AB}I_{A}⊗M_{k}^{\left(B\right)})}{\mathrm{Tr}\left(\sigma_{AB}I_{A}⊗M_{k}^{\left(B\right)}\right)+\mathrm{Tr}\left({\bar{\sigma }}_{AB}{\bar{I}}_{A}⊗M_{k}^{\left(B\right)}\right)}\frac{{\mathrm{Tr}}_{B}\left(\sigma_{AB}I_{A}⊗M_{k}^{\left(B\right)}\right)}{\mathrm{Tr}(\sigma_{AB}I_{A}⊗M_{k}^{\left(B\right)})}\\ & & \;\;\;\;\;\;\;\;\;\;\;\;\;\;\;\;\;\;\;\;\;\;\;\;\;\;\;\;\;\;\;\;\;\;\;\;\;\;\;\;\;\;\;\;\;\;\;\;+\frac{\mathrm{Tr}({\bar{\sigma }}_{AB}{\bar{I}}_{A}⊗M_{k}^{\left(B\right)})}{\mathrm{Tr}\left(\sigma_{AB}I_{A}⊗M_{k}^{\left(B\right)}\right)+\mathrm{Tr}\left({\bar{\sigma }}_{AB}{\bar{I}}_{A}⊗M_{k}^{\left(B\right)}\right)}\frac{{\mathrm{Tr}}_{B}\left({\bar{\sigma }}_{AB}{\bar{I}}_{A}⊗M_{k}^{\left(B\right)}\right)}{\mathrm{Tr}({\bar{\sigma }}_{AB}{\bar{I}}_{A}⊗M_{k}^{\left(B\right)})})\\ & & =S\left(\frac{\mathrm{Tr}(\sigma_{AB}I_{A}⊗M_{k}^{\left(B\right)})}{\mathrm{Tr}\left(\sigma_{AB}I_{A}⊗M_{k}^{\left(B\right)}\right)+\mathrm{Tr}\left({\bar{\sigma }}_{AB}{\bar{I}}_{A}⊗M_{k}^{\left(B\right)}\right)}\sigma_{A|k}^{\prime }+\frac{\mathrm{Tr}({\bar{\sigma }}_{AB}{\bar{I}}_{A}⊗M_{k}^{\left(B\right)})}{\mathrm{Tr}\left(\sigma_{AB}I_{A}⊗M_{k}^{\left(B\right)}\right)+\mathrm{Tr}\left({\bar{\sigma }}_{AB}{\bar{I}}_{A}⊗M_{k}^{\left(B\right)}\right)}{{\bar{\sigma }}^{\prime }}_{A|k}\right)\\ & & \geq \frac{\mathrm{Tr}(\sigma_{AB}I_{A}⊗M_{k}^{\left(B\right)})}{\mathrm{Tr}\left(\sigma_{AB}I_{A}⊗M_{k}^{\left(B\right)}\right)+\mathrm{Tr}\left({\bar{\sigma }}_{AB}{\bar{I}}_{A}⊗M_{k}^{\left(B\right)}\right)}S\left(\sigma_{A|k}^{\prime }\right)\\ & & \;\;\;\;\;\;\;\;\;\;\;\;\;\;\;\;\;\;\;\;\;\;\;\;\;\;\;\;\;\;\;\;\;\;\;\;\;\;\;\;\;\;\;\;\;\;\;\;+\frac{\mathrm{Tr}({\bar{\sigma }}_{AB}{\bar{I}}_{A}⊗M_{k}^{\left(B\right)})}{\mathrm{Tr}\left(\sigma_{AB}I_{A}⊗M_{k}^{\left(B\right)}\right)+\mathrm{Tr}\left({\bar{\sigma }}_{AB}{\bar{I}}_{A}⊗M_{k}^{\left(B\right)}\right)}S\left({{\bar{\sigma }}^{\prime }}_{A|k}\right). \end{array}$$

Hence, conditional entropy satisfies

$$\begin{array}{c} \sum_{k=1}^{N}p_{k}S\left(\rho_{A|k}\right)\geq \sum_{k=1}^{N}\mathrm{Tr}\left(\sigma_{AB}I_{A}⊗M_{k}^{\left(B\right)}\right)S\left(\sigma_{A|k}^{\prime }\right)+\sum_{k=1}^{N}\mathrm{Tr}\left({\bar{\sigma }}_{AB}I_{A}⊗M_{k}^{\left(B\right)}\right)S\left({\bar{\sigma }}_{A|k}^{\prime }\right). \end{array}$$

Also, minimum of conditional entropy also satisfies

$$\begin{array}{ccc} \min_{\left\{M_{k}^{\left(B\right)}\right\}}\sum_{k=1}^{N}p_{k}S\left(\rho_{A|k}\right) & \geq & \underset{\mathrm{Problem}\;I}{\underset{︸}{\min_{\left\{M_{k}^{\left(B\right)}\right\}}\left[\left(\mathrm{Tr}\sigma_{AB}\right)\sum_{k=1}^{N}p_{k}^{\prime }S\left(\sigma_{A|k}^{\prime }\right)+\left(\mathrm{Tr}{\bar{\sigma }}_{AB}\right)\sum_{k=1}^{N}{\bar{p}}_{k}^{\prime }S\left({\bar{\sigma }}_{A|k}^{\prime }\right)\right]}}\\ & \geq & \underset{\mathrm{Problem}\;\mathrm{II}}{\underset{︸}{\left(\mathrm{Tr}\sigma_{AB}\right)\min_{\left\{M_{k}^{\left(B\right)}\right\}}\sum_{k=1}^{N}p_{k}^{\prime }S\left(\sigma_{A|k}^{\prime }\right)+\left(\mathrm{Tr}{\bar{\sigma }}_{AB}\right)\min_{\left\{{\bar{M}}_{k}^{\left(B\right)}\right\}}\sum_{k=1}^{N}{\bar{p}}_{k}^{\prime }S\left({{\bar{\sigma }}^{\prime }}_{A|k}\right)}}\\ & = & \left(\mathrm{Tr}\sigma_{AB}\right)E_{f}\left(\sigma_{AE}^{\prime }\right)+\left(\mathrm{Tr}{\bar{\sigma }}_{AB}\right)E_{f}\left({\bar{\sigma }}_{A\bar{E}}^{\prime }\right). \end{array}$$

This inequality consists of two optimization problems (Problem I and Problem II). The Problem I contains additional equality constraint $M_{k}^{\left(B\right)}={\bar{M}}_{k}^{\left(B\right)}\left(\forall k\right)$, but this constraint is not included in the Problem II. Therefore, minimum of the Problem I is not smaller than that of the Problem II. Finally, we obtain the lower bound of quantum discord as

$$\begin{array}{ccc} D_{B}\left(\rho_{AB}\right) & = & S\left(\rho_{B}\right)-S\left(\rho_{AB}\right)+\min_{\left\{M_{k}^{\left(B\right)}\right\}}\sum_{k=1}^{N}p_{k}S\left(\rho_{A|k}\right)\\ & \geq & S\left(\rho_{B}\right)-S\left(\rho_{AB}\right)+\left(\mathrm{Tr}\sigma_{AB}\right)E_{f}\left(\sigma_{AE}^{\prime }\right)+\left(\mathrm{Tr}{{\bar{\sigma }}^{\prime }}_{AB}\right)E_{f}\left({{\bar{\sigma }}^{\prime }}_{A\bar{E}}\right)\\ & = & \left(\mathrm{Tr}\sigma_{AB}\right)D_{B}\left(\sigma_{AB}^{\prime }\right)+\left(\mathrm{Tr}{\bar{\sigma }}_{AB}\right)D_{B}\left({\bar{\sigma }}_{AB}^{\prime }\right)=W_{B}\left(\rho_{AB}\right). \end{array}$$

A.3 Deriving the relationship between discord $D_{AB}\left(\rho_{AB}\right)$ and generalized witness $W_{AB}\left(\rho_{AB}\right)$.

In this case, derivation is more straightforward. Substituting $D_{A}\left(\rho_{AB}\right)$ and $D_{B}\left(\rho_{AB}\right)$ into $D_{AB}\left(\rho_{AB}\right)$, we obtain

$$\begin{array}{ccc} & & D_{AB}\left(\rho_{AB}\right)=\sqrt{D_{A}\left(\rho_{AB}\right)D_{B}\left(\rho_{AB}\right)}\\ & & \geq \sqrt{\left(\mathrm{Tr}\sigma_{AB}\right)D_{A}\left(\sigma_{AB}^{\prime }\right)+\left(\mathrm{Tr}{\bar{\sigma }}_{AB}\right)D_{A}\left({\bar{\sigma }}_{AB}^{\prime }\right)}\times \sqrt{\left(\mathrm{Tr}\sigma_{AB}\right)D_{B}\left(\sigma_{AB}^{\prime }\right)+\left(\mathrm{Tr}{\bar{\sigma }}_{AB}\right)D_{B}\left({\bar{\sigma }}_{AB}^{\prime }\right)}\\ & & ={∥\left[\sqrt{\left(\mathrm{Tr}\sigma_{AB}\right)D_{A}\left(\sigma_{AB}^{\prime }\right)},\sqrt{\left(\mathrm{Tr}{\bar{\sigma }}_{AB}\right)D_{A}({\bar{\sigma }}_{AB}^{\prime }}\right]∥}_{2}{∥\left[\sqrt{\left(\mathrm{Tr}\sigma_{AB}\right)D_{B}\left(\sigma_{AB}^{\prime }\right)},\sqrt{\left(\mathrm{Tr}{\bar{\sigma }}_{AB}\right)D_{B}({\bar{\sigma }}_{AB}^{\prime }}\right]∥}_{2}\\ & & \geq \left[\sqrt{\left(\mathrm{Tr}\sigma_{AB}\right)D_{A}\left(\sigma_{AB}^{\prime }\right)},\sqrt{\left(\mathrm{Tr}{\bar{\sigma }}_{AB}\right)D_{A}\left({\bar{\sigma }}_{AB}^{\prime }\right)}\right]\cdot \left[\sqrt{\left(\mathrm{Tr}\sigma_{AB}\right)D_{B}\left(\sigma_{AB}^{\prime }\right)},\sqrt{\left(\mathrm{Tr}{\bar{\sigma }}_{AB}\right)D_{B}\left({\bar{\sigma }}_{AB}^{\prime }\right)}\right]\\ & & =\left(\mathrm{Tr}\sigma_{AB}\right)\sqrt{D_{A}\left(\sigma_{AB}^{\prime }\right)D_{B}\left(\sigma_{AB}^{\prime }\right)}+\left(\mathrm{Tr}{\bar{\sigma }}_{AB}\right)\sqrt{D_{A}\left({\bar{\sigma }}_{AB}^{\prime }\right)D_{B}\left({\bar{\sigma }}_{AB}^{\prime }\right)}\\ & & =\left(\mathrm{Tr}\sigma_{AB}\right)D_{AB}\left(\sigma_{AB}^{\prime }\right)+\left(\mathrm{Tr}{\bar{\sigma }}_{AB}\right)D_{AB}\left({\bar{\sigma }}_{AB}^{\prime }\right)=W_{AB}\left(\rho_{AB}\right). \end{array}$$

In second inequality, the Cauchy Schwarz inequality is used: ${\left||u|\right|}_{2}{\left||v|\right|}_{2}\geq u\cdot v$ (for all real vectors u,v).

## Appendix B. Deriving the Generalized RQC Discrepancy

In this Appendix, we derive the generalized RQC discrepancy. Two positive semidefinite operators $\sigma_{AB}$ and ${\bar{\sigma }}_{AB}$ in Equation (A1) are defined over sub-Hilbert space H and $\bar{H}$. Therefore, the entire Hilbert space $H⊕\bar{H}$ consists of orthonormal basis ${\left\{|v_{i}^{\left(A\right)}\rangle ⊗|v_{j}^{\left(B\right)}\rangle \right\}}_{i,j}$ and ${\left\{|{\bar{v}}_{i}^{\left(A\right)}\rangle ⊗|{\bar{v}}_{j}^{\left(B\right)}\rangle \right\}}_{i,j}$. Here, sub-Hilbert space H($\bar{H}$) is composed of ${\left\{|v_{i}^{\left(A\right)}\rangle ⊗|v_{j}^{\left(B\right)}\rangle \right\}}_{i,j}$(${\left\{|{\bar{v}}_{i}^{\left(A\right)}\rangle ⊗|{\bar{v}}_{j}^{\left(B\right)}\rangle \right\}}_{i,j}$). Using this property of Hilbert space, we can evaluate von Neumann entropy of the incoherent state $\rho_{PQ}$ as [33]

$$\begin{array}{ccc} & & S(\rho_{PQ})\\ & & =-\sum_{i,j}\langle v_{i}^{\left(A\right)}⊗v_{j}^{\left(B\right)}\left|\rho_{AB}\right|v_{i}^{\left(A\right)}⊗v_{j}^{\left(B\right)}\rangle \log_{2}\langle v_{i}^{\left(A\right)}⊗v_{j}^{\left(B\right)}\left|\rho_{AB}\right|v_{i}^{\left(A\right)}⊗v_{j}^{\left(B\right)}\rangle \\ & & -\sum_{i,j}\langle {\bar{v}}_{i}^{\left(A\right)}⊗{\bar{v}}_{j}^{\left(B\right)}\left|\rho_{AB}\right|{\bar{v}}_{i}^{\left(A\right)}⊗{\bar{v}}_{j}^{\left(B\right)}\rangle \log_{2}\langle {\bar{v}}_{i}^{\left(A\right)}⊗{\bar{v}}_{j}^{\left(B\right)}\left|\rho_{AB}\right|{\bar{v}}_{i}^{\left(A\right)}⊗{\bar{v}}_{j}^{\left(B\right)}\rangle \\ & & =-\left(\mathrm{Tr}\sigma_{AB}\right)\sum_{i,j}\langle v_{i}^{\left(A\right)}⊗v_{j}^{\left(B\right)}\left|\frac{\sigma_{AB}}{\mathrm{Tr}\sigma_{AB}}\right|v_{i}^{\left(A\right)}⊗v_{j}^{\left(B\right)}\rangle \left\{\log_{2}\langle v_{i}^{\left(A\right)}⊗v_{j}^{\left(B\right)}\left|\frac{\sigma_{AB}}{\mathrm{Tr}\sigma_{AB}}\right|v_{i}^{\left(A\right)}⊗v_{j}^{\left(B\right)}\rangle +\log_{2}\left(\mathrm{Tr}\sigma_{AB}\right)\right\}\\ & & -\left(\mathrm{Tr}{\bar{\sigma }}_{AB}\right)\sum_{i,j}\langle {\bar{v}}_{i}^{\left(A\right)}⊗{\bar{v}}_{j}^{\left(B\right)}\left|\frac{{\bar{\sigma }}_{AB}}{\mathrm{Tr}{\bar{\sigma }}_{AB}}\right|{\bar{v}}_{i}^{\left(A\right)}⊗{\bar{v}}_{j}^{\left(B\right)}\rangle \left\{\log_{2}\langle {\bar{v}}_{i}^{\left(A\right)}⊗{\bar{v}}_{j}^{\left(B\right)}\left|\frac{{\bar{\sigma }}_{AB}}{\mathrm{Tr}{\bar{\sigma }}_{AB}}\right|{\bar{v}}_{i}^{\left(A\right)}⊗{\bar{v}}_{j}^{\left(B\right)}\rangle +\log_{2}\left(\mathrm{Tr}{\bar{\sigma }}_{AB}\right)\right\}\\ & & =\left(\mathrm{Tr}\sigma_{AB}\right)S\left(\sigma_{PQ}^{\prime }\right)+\left(\mathrm{Tr}{\bar{\sigma }}_{AB}\right)S\left({\bar{\sigma }}_{PQ}^{\prime }\right)-\left(\mathrm{Tr}\sigma_{AB}\right)\log_{2}\left(\mathrm{Tr}\sigma_{AB}\right)-\left(\mathrm{Tr}{\bar{\sigma }}_{AB}\right)\log_{2}\left(\mathrm{Tr}{\bar{\sigma }}_{AB}\right). \end{array}$$

Also, the RQC of $\rho_{AB}$ is evaluated as

$$\begin{array}{ccc} & C_{\mathrm{rel}.\mathrm{ent}} & \left(\rho_{AB},\rho_{PQ}\right)\\ & = & S\left(\rho_{PQ}\right)-S\left(\rho_{AB}\right)\\ & = & \left(\mathrm{Tr}\sigma_{AB}\right)\left\{S\left(\sigma_{PQ}^{\prime }\right)-S\left(\sigma_{AB}^{\prime }\right)\right\}+\left(\mathrm{Tr}{\bar{\sigma }}_{AB}\right)\left\{S\left({\bar{\sigma }}_{PQ}^{\prime }\right)-S\left({\bar{\sigma }}_{AB}^{\prime }\right)\right\}\\ & = & \left(\mathrm{Tr}\sigma_{AB}\right)C_{\mathrm{rel}.\mathrm{ent}}\left(\sigma_{AB}^{\prime },\sigma_{PQ}^{\prime }\right)+\left(\mathrm{Tr}{\bar{\sigma }}_{AB}\right)C_{\mathrm{rel}.\mathrm{ent}}\left({\bar{\sigma }}_{AB}^{\prime },{\bar{\sigma }}_{PQ}^{\prime }\right). \end{array}$$

Next, we evaluate the RQC of system *A* and *B*. First, we consider the partial state $\rho_{A}$:

$$\begin{array}{c} \rho_{A}=q_{1}r_{1}|s_{1}\rangle \langle s_{1}|+q_{2}r_{2}|s_{2}\rangle \langle s_{2}|+q_{1}{\bar{r}}_{1}|{\bar{s}}_{1}\rangle \langle {\bar{s}}_{1}|+q_{2}{\bar{r}}_{2}|{\bar{s}}_{2}\rangle \langle {\bar{s}}_{2}|=\sigma_{A}+{\bar{\sigma }}_{A}. \end{array}$$

As the supports of $\sigma_{A}$ and ${\bar{\sigma }}_{A}$ are orthogonal, von Neumann entropy of $\rho_{A}$ is expressed as

$$\begin{array}{ccc} S\left(\rho_{A}\right)=S\left(\sigma_{A}+{\bar{\sigma }}_{A}\right) & = & \left(\mathrm{Tr}\sigma_{A}\right)S\left(\sigma_{A}^{\prime }\right)+\left(\mathrm{Tr}{\bar{\sigma }}_{A}\right)S\left({\bar{\sigma }}_{A}^{\prime }\right)-\left(\mathrm{Tr}\sigma_{A}\right)\log_{2}\left(\mathrm{Tr}\sigma_{A}\right)-\left(\mathrm{Tr}{\bar{\sigma }}_{A}\right)\log_{2}\left(\mathrm{Tr}{\bar{\sigma }}_{A}\right)\\ & = & \left(\mathrm{Tr}\sigma_{A}\right)S\left(\sigma_{A}^{\prime }\right)+\left(\mathrm{Tr}{\bar{\sigma }}_{A}\right)S\left({\bar{\sigma }}_{A}^{\prime }\right)-\left(\mathrm{Tr}\sigma_{AB}\right)\log_{2}\left(\mathrm{Tr}\sigma_{A}\right)-\left(\mathrm{Tr}{\bar{\sigma }}_{AB}\right)\log_{2}\left(\mathrm{Tr}{\bar{\sigma }}_{A}\right). \end{array}$$

As the partial state $\rho_{P}$ is given as $\rho_{P}=\sigma_{P}⊕{\bar{\sigma }}_{P}$, von Neumann entropy of $\rho_{P}$ is also expressed as

$$\begin{array}{ccc} S(\rho_{P}) & = & -\sum_{i}\langle v_{i}^{\left(A\right)}\left|\rho_{A}\right|v_{i}^{\left(A\right)}\rangle \log_{2}\langle v_{i}^{\left(A\right)}\left|\rho_{A}\right|v_{i}^{\left(A\right)}\rangle -\sum_{i}\langle {\bar{v}}_{i}^{\left(A\right)}\left|\rho_{A}\right|{\bar{v}}_{i}^{\left(A\right)}\rangle \log_{2}\langle {\bar{v}}_{i}^{\left(A\right)}\left|\rho_{A}\right|{\bar{v}}_{i}^{\left(A\right)}\rangle \\ & = & -\left(\mathrm{Tr}\sigma_{A}\right)\sum_{i}\langle v_{i}^{\left(A\right)}\left|\frac{\sigma_{A}}{\mathrm{Tr}\sigma_{A}}\right|v_{i}^{\left(A\right)}\rangle \left\{\log_{2}\langle v_{i}^{\left(A\right)}\left|\frac{\sigma_{A}}{\mathrm{Tr}\sigma_{A}}\right|v_{i}^{\left(A\right)}\rangle +\log_{2}\left(\mathrm{Tr}\sigma_{A}\right)\right\}\\ & - & \left(\mathrm{Tr}{\bar{\sigma }}_{A}\right)\sum_{i}\langle {\bar{v}}_{i}^{\left(A\right)}\left|\frac{{\bar{\sigma }}_{A}}{\mathrm{Tr}{\bar{\sigma }}_{A}}\right|{\bar{v}}_{i}^{\left(A\right)}\rangle \left\{\log_{2}\langle {\bar{v}}_{i}^{\left(A\right)}\left|\frac{{\bar{\sigma }}_{A}}{\mathrm{Tr}{\bar{\sigma }}_{A}}\right|{\bar{v}}_{i}^{\left(A\right)}\rangle +\log_{2}\left(\mathrm{Tr}{\bar{\sigma }}_{A}\right)\right\}\\ & = & \left(\mathrm{Tr}\sigma_{A}\right)S\left(\sigma_{P}^{\prime }\right)+\left(\mathrm{Tr}{\bar{\sigma }}_{A}\right)S\left({\bar{\sigma }}_{P}^{\prime }\right)-\left(\mathrm{Tr}\sigma_{A}\right)\log_{2}\left(\mathrm{Tr}\sigma_{A}\right)-\left(\mathrm{Tr}{\bar{\sigma }}_{A}\right)\log_{2}\left(\mathrm{Tr}{\bar{\sigma }}_{A}\right)\\ & = & \left(\mathrm{Tr}\sigma_{AB}\right)S\left(\sigma_{P}^{\prime }\right)+\left(\mathrm{Tr}{\bar{\sigma }}_{AB}\right)S\left({\bar{\sigma }}_{P}^{\prime }\right)-\left(\mathrm{Tr}\sigma_{AB}\right)\log_{2}\left(\mathrm{Tr}\sigma_{A}\right)-\left(\mathrm{Tr}{\bar{\sigma }}_{AB}\right)\log_{2}\left(\mathrm{Tr}{\bar{\sigma }}_{A}\right). \end{array}$$

Therefore, the RQC of system *A* is derived as

$$\begin{array}{ccc} C_{\mathrm{rel}.\mathrm{ent}}\left(\rho_{A},\rho_{P}\right) & = & S\left(\rho_{P}\right)-S\left(\rho_{A}\right)\\ & = & \left(\mathrm{Tr}\sigma_{AB}\right)\left\{S\left(\sigma_{P}^{\prime }\right)-S\left(\sigma_{A}^{\prime }\right)\right\}+\left(\mathrm{Tr}{\bar{\sigma }}_{AB}\right)\left\{S\left({\bar{\sigma }}_{P}^{\prime }\right)-S\left({\bar{\sigma }}_{A}^{\prime }\right)\right\}\\ & = & \left(\mathrm{Tr}\sigma_{AB}\right)C_{\mathrm{rel}.\mathrm{ent}}\left(\sigma_{A}^{\prime },\sigma_{P}^{\prime }\right)+\left(\mathrm{Tr}{\bar{\sigma }}_{AB}\right)C_{\mathrm{rel}.\mathrm{ent}}\left({\bar{\sigma }}_{A}^{\prime },{\bar{\sigma }}_{P}^{\prime }\right). \end{array}$$

Finally, we derive generalized RQC discrepancy $\delta_{A}\left(\rho_{AB}\right)$ as

$$\begin{array}{ccc} & & \delta_{A}\left(\rho_{AB}\right)=C_{\mathrm{rel}.\mathrm{ent}}\left(\rho_{AB},\rho_{PQ}\right)-C_{\mathrm{rel}.\mathrm{ent}}\left(\rho_{A},\rho_{P}\right)\\ & & =\{\left(\mathrm{Tr}\sigma_{AB}\right)C_{\mathrm{rel}.\mathrm{ent}}\left(\sigma_{AB}^{\prime },\sigma_{PQ}^{\prime }\right)+\left(\mathrm{Tr}{\bar{\sigma }}_{AB}\right)C_{\mathrm{rel}.\mathrm{ent}}\left({\bar{\sigma }}_{AB}^{\prime },{\bar{\sigma }}_{PQ}^{\prime }\right)\}\\ & & \;\;\;\;\;\;\;\;\;\;\;\;\;\;\;\;\;\;\;\;\;\;\;\;-\{\left(\mathrm{Tr}\sigma_{AB}\right)C_{\mathrm{rel}.\mathrm{ent}}\left(\sigma_{A}^{\prime },\sigma_{P}^{\prime }\right)+\left(\mathrm{Tr}{\bar{\sigma }}_{AB}\right)C_{\mathrm{rel}.\mathrm{ent}}\left({\bar{\sigma }}_{A}^{\prime },{\bar{\sigma }}_{P}^{\prime }\right)\}\\ & & =\left(\mathrm{Tr}\sigma_{AB}\right)\left\{C_{\mathrm{rel}.\mathrm{ent}}\left(\sigma_{AB}^{\prime },\sigma_{PQ}^{\prime }\right)-C_{\mathrm{rel}.\mathrm{ent}}\left(\sigma_{A}^{\prime },\sigma_{P}^{\prime }\right)\right\}\\ & & \;\;\;\;\;\;\;\;\;\;\;\;\;\;\;\;\;\;\;\;\;\;\;\;+\left(\mathrm{Tr}{\bar{\sigma }}_{AB}\right)\left\{C_{\mathrm{rel}.\mathrm{ent}}\left({\bar{\sigma }}_{AB}^{\prime },{\bar{\sigma }}_{PQ}^{\prime }\right)-C_{\mathrm{rel}.\mathrm{ent}}\left({\bar{\sigma }}_{A}^{\prime },{\bar{\sigma }}_{P}^{\prime }\right)\right\}\\ & & =\left(\mathrm{Tr}\sigma_{AB}\right)\delta_{A}\left(\sigma_{AB}^{\prime }\right)+\left(\mathrm{Tr}{\bar{\sigma }}_{AB}\right)\delta_{A}\left({\bar{\sigma }}_{AB}^{\prime }\right). \end{array}$$

In a similar manner, the generalized RQC discrepancy $\delta_{B}\left(\rho_{AB}\right)$ is also derived as

$$\begin{array}{c} \delta_{B}\left(\rho_{AB}\right)=\left(\mathrm{Tr}\sigma_{AB}\right)\delta_{B}\left(\sigma_{AB}^{\prime }\right)+\left(\mathrm{Tr}{\bar{\sigma }}_{AB}\right)\delta_{B}\left({\bar{\sigma }}_{AB}^{\prime }\right). \end{array}$$

## References

[1] Ivanovic I.D. (1987). How to differentiate between non-orthogonal states. Phys. Lett. A.

[2] Dieks D. (1988). Overlap and distinguishability of quantum states. Phys. Lett. A.

[3] Peres A. (1988). How to differentiate between non-orthogonal states. Phys. Lett. A.

[4] Jaeger G., Shimony A. (1995). Optimal distinction between two non-orthogonal quantum states. Phys. Lett. A.

[5] Pang S., Wu S. (2009). Optimum unambiguous discrimination of linearly independent pure states. Phys. Rev. A.

[6] Bergou J.A., Futschik U., Feldman E. (2012). Optimal Unambiguous Discrimination of Pure Quantum States. Phys. Rev. Lett..

[7] Ha D., Kwon Y. (2015). Analysis of optimal unambiguous discrimination of three pure quantum states. Phys. Rev. A.

[8] Nielson M.A., Chuang I.L. (2010). Quantum Computation and Quantum Information: 10th Anniversary Edition.

[9] Zhou X.-F., Liu Q., Zheng Y.-S., Guo G.-C. (2007). Physical accessible transformation on a finite number of quantum states. Phys. Rev. A.

[10] Bergou J.A., Feldman E., Hillery M. (2013). Extracting Information from a Qubit by Multiple Observers: Towards a Theory of Sequential State Discrimination. Phys. Rev. Lett..

[11] Pang C.-Q., Zhang F.-L., Liang M.-L. (2013). Sequential state discrimination and requirement of quantum dissonance. Phys. Rev. A.

[12] Solis-Prosser M.A., Gonzalez P., Fuenzalida J., Gomez S., Xavier G.B., Delgado A., Lima G. (2016). Experimental multiparty SSD. Phys. Rev. A.

[13] Hillery M., Mimih J. (2016). Sequential discrimination of qudits by multiple observers. J. Phys. A Math. Theor..

[14] Namkung M., Kwon Y. (2017). Optimal sequential state discrimination between two mixed quantum states. Phys. Rev. A.

[15] Zhang J.-H., Zhang F.-L., Liang M.-L. (2018). Sequential state discrimination with quantum correlation. Quant. Inf. Process..

[16] Namkung M., Kwon Y. (2018). Analysis of Optimal Sequential State Discrimination for Linearly Independent Pure Quantum States. Sci. Rep..

[17] Namkung M., Kwon Y. (2018). Sequential state discrimination of coherent states. Sci. Rep..

[18] Namkung M., Kwon Y. (2020). Generalized sequential state discrimination for multiparty QKD and its optical implementation. Sci. Rep..

[19] Li B., Fei S.-M., Wang Z.-X., Fan H. (2012). Assisted state discrimination without entanglement. Phys. Rev. A.

[20] Roa L., Retamal J.C., Alid-Vaccarezza M. (2011). Dissonance is Required for Assisted Optimal State Discrimination. Phys. Rev. Lett..

[21] Xu L.F., Zhang F.L., Liang M.L., Chen J.L. (2014). Assisted optimal state discrimination without entanglement. EPL.

[22] Zhang F.-L., Chen J.-L., Kwek L.C., Vedral V. (2013). Requirement of Dissonance in Assisted Optimal State Discrimination. Sci. Rep..

[23] Baumgratz T., Cramer M., Plenio M.B. (2014). Quantifying Coherence. Phys. Rev. Lett..

[24] Bera M.N., Qureshi T., Siddiqui M.A., Pati A.K. (2015). Duality of quantum coherence and path distinguishability. Phys. Rev. A.

[25] Bagan E., Bergou J.A., Cottrell S.S., Hillery M. (2016). Relations between Coherence and Path Information. Phys. Rev. Lett..

[26] Bagan E., Calsamiglia J., Bergou J.A., Hillery M. (2018). Duality Games and Operational Duality Relation. Phys. Rev. Lett..

[27] Hillery M. (2016). Coherence as a resource in decision problems: The Deutsch-Jozsa algorithm and a variation. Phys. Rev. A.

[28] Shi H.-L., Liu S.-Y., Wang X.-H., Yang W.-L., Yang Z.-Y., Fan H. (2017). Coherence depletion in the Grover quantum search algorithm. Phys. Rev. A.

[29] Pan M., Qiu D. (2019). Operator coherence dynamics in Grover’s quantum search algorithm. Phys. Rev. A.

[30] Liu Y.-C., Shang J., Zhang X. (2019). Coherence Depletion in Quantum Algorithms. Entropy.

[31] Ma J., Hakande A., Yuan X., Ma X. (2019). Coherence as a resource for source-independent quantum random-number generation. Phys. Rev. A.

[32] Namkung M., Kwon Y. (2020). Coherence and Entanglement Dynamics in Training Quantum Perceptron. Entropy.

[33] Hu M.-L., Fan H. (2017). Relative quantum coherence, incompatibility, and quantum correlations of states. Phys. Rev. A.

[34] Ollivier H., Jurek W.H. (2001). Quantum Discord: A Measure of The Quantumness of Correlation. Phys. Rev. Lett..

[35] Hamieh S., Kobes R., Zaraket H. (2004). Positive-operator-valued measure optimization of classical correlations. Phys. Rev. A.

[36] Luo S. (2008). Quantum discord for two-qubit systems. Phys. Rev. A.

[37] Ali M., Rau A.R.P., Alber G. (2010). Quantum discord for two-qubit *X* states. Phys. Rev. A.

[38] Chen Q., Zhang C., Yu S., Yi X.X., Oh C.H. (2011). Quantum discord of two-qubit *X* states. Phys. Rev. A.

[39] Huang Y. (2013). Quantum discord for two-qubit *X* states: Analytical formula with very small worst-case error. Phys. Rev. A.

[40] Namkung M., Chang J., Shin J., Kwon Y. (2015). Revisiting Quantum Discord for Two-Qubit X States: The Error Bound to an Analytical Formula. Int. J. Theor. Phys..

[41] Modi K., Paterek T., Son W., Vedral V., Williamson M. (2010). Unified View of Quantum and Classical Correlations. Phys. Rev. Lett..

[42] Brask J.B., Martin A., Esposito W., Houlmann R., Bowles J., Zbinden H., Brunner N. (2017). Megahertz-Rate Semi-Device-Independent Quantum Random Number Generators Based on Unambiguous State Discrimination. Phys. Rev. Appl..

[43] Bennett C.H., Brassard G. Quantum cryptography: Public Key distribution and coin tossing. Proceedings of the International Conference on Computers, Systems and Signal Processing.

[44] Bennett C.H. (1992). Quantum Cryptography Using Any Two Nonorthogonal States. Phys. Rev. Lett..

[45] Ha D., Kwon Y. (2018). A minimal set of measurements for qudit-state tomography based on unambiguous discrimination. Quant. Inf. Process..

[46] Henderson L., Vedral V. (2001). Classical, quantum and total correlations. J. Phys. A Math. Gen..

[47] Duan L.-M., Guo G.-C. (1998). Probabilistic cloning and identification of linearly independent quantum states. Phys. Rev. Lett..

[48] Koashi M., Winter A. (2004). Monogamy of quantum entanglement and other correlations. Phys. Rev. A.

[49] Rudolph T., Spekken R.W., Turner P.S. (2003). Unambiguous discrimination of mixed states. Phys. Rev. A.

[50] Raynal P., Lutkenhaus N., van Enk S.J. (2003). Reduction theorems for optimal unambiguous state discrimination of density matrices. Phys. Rev. A.

[51] Herzog U. (2007). Optimum unambiguous discrimination of two mixed states and application to a class of similar states. Phys. Rev. A.

[52] Helstrom C.W. (1976). Quantum Detection and Estimation Theory.

[53] Bae J. (2013). Structure of minimum-error quantum state discrimination. New J. Phys..

[54] Ha D., Kwon Y. (2013). Complete analysis of three-qubit mixed-state discrimination. Phys. Rev. A.

[55] Ha D., Kwon Y. (2014). Discriminating *N*-qudit states using geometric structure. Phys. Rev. A.

[56] Namkung M., Kwon Y. (2019). Almost minimum error discrimination of *N*-ary weak coherent states by Jaynes-Cummings Hamiltonian dynamics. Sci. Rep..

[57] Kim J., Ha D., Kwon Y. (2019). Uniqueness of Minimax Strategy in View of Minimum Error Discrimination of Two Quantum States. Entropy.

[58] Han R., Leuchs G., Bergou J.A. (2020). The Helstrom measurement: A nondestructive implementation. Phys. Rev. A.

[59] Ha D., Kwon Y. (2017). An optimal discrimination of two mixed qubit states with a fixed rate of inconclusive results. Quant. Inf. Process..

[60] Zhang W.-H., Ren G. (2018). State discrimination of two pure states with a fixed rate of inconclusive answer. J. Mod. Opt..

[61] Du Y., Hsieh M.-H., Liu T., Tao D. (2018). Implementable Quantum Classifier for Nonlinear Data. arXiv.

[62] Wootters W.K. (1998). Entanglement of formation of an arbitrary state of two qubits. Phys. Rev. Lett..

